# Super-Resolution of Magnetic Resonance Images via Convex Optimization with Local and Global Prior Regularization and Spectrum Fitting

**DOI:** 10.1155/2018/9262847

**Published:** 2018-09-02

**Authors:** Naoki Kawamura, Tatsuya Yokota, Hidekata Hontani

**Affiliations:** Nagoya Institute of Technology, Gokiso-cho, Showa-ku, Nagoya-shi, Aichi, Japan

## Abstract

Given a low-resolution image, there are many challenges to obtain a super-resolved, high-resolution image. Many of those approaches try to simultaneously upsample and deblur an image in signal domain. However, the nature of the super-resolution is to restore high-frequency components in frequency domain rather than upsampling in signal domain. In that sense, there is a close relationship between super-resolution of an image and extrapolation of the spectrum. In this study, we propose a novel framework for super-resolution, where the high-frequency components are theoretically restored with respect to the frequency fidelities. This framework helps to introduce multiple simultaneous regularizers in both signal and frequency domains. Furthermore, we propose a new super-resolution model where frequency fidelity, low-rank (LR) prior, low total variation (TV) prior, and boundary prior are considered at once. The proposed method is formulated as a convex optimization problem which can be solved by the alternating direction method of multipliers. The proposed method is the generalized form of the multiple super-resolution methods such as TV super-resolution, LR and TV super-resolution, and the Gerchberg method. Experimental results show the utility of the proposed method comparing with some existing methods using both simulational and practical images.

## 1. Introduction

Magnetic resonance (MR) imaging is one of the most important methods for observing three-dimensional (3D) soft tissues with high contrast (e.g., [1–3]). However in order to assure sufficiently high signal-to-noise ratio (SNR), MR images often have anisotropic spatial resolution: The spatial resolution along the through-slice direction is lower than the resolution along the in-plane direction. The spatial resolution along the through-slice direction is mainly determined by the slice thickness, and there is a trade-off between the slice thickness and the SNR of MR images. Increase of the slice thickness would degrade the spatial resolution along the through-slice direction, though the SNR of each slice image would be improved by the increase of the slice thickness because the quantity of hydrogen nuclei included in the measured slice increases and the magnitude of the signals emitted by the hydrogen nuclei also increases. This is a reason why slice thickness is often set as thick as several times the pixel size and the spatial resolution along the through-slice direction is lower than that of slice images.

Super-resolution techniques (e.g., [1, 2, 4–6]) can restore detailed patterns unobservable in given images and can be used for improving spatial resolutions of MR images. In this article we at first focus on a conventional super-resolution algorithm, which was proposed by Gerchberg [7]. The algorithm improves the spatial resolution of a given image by using the prior knowledge of an outer boundary of the target and of the measurable frequency range of a target spatial pattern of the given image. The physical meaning of the prior knowledge used in the algorithm is well understandable and the algorithm can be applied to MR images straightforwardly. The method iteratively improves the spatial resolution of a given image and it is proved that the restored image converges toward the true solution when the prior knowledge and the reality are consistent [8]. In practice, the results obtained by the Gerchberg algorithm may be affected by ringing artifacts [9–11] and are degraded by measurement noises. Reference [12] formulated the algorithm in a signal-extrapolation framework and the method is now called the Papoulis-Gerchberg algorithm [9, 10, 12].

The Gerchberg algorithm is essentially the same with the projection onto convex sets (POCS) method, which was later defined by Youla [8, 13]. Many POCS methods have been proposed where the super-resolution problem is solved by iteratively projecting a given image onto two or more convex sets, each of which represents each of the introduced constraints on the reconstructed image (e.g., [14–18]). The constraints to be introduced vary depending on the available prior knowledge of images: The knowledge that can be employed by the POCS method includes the range of pixel values [15, 19], the fidelity of the data [15, 17, 20], and nonnegativity [16, 17]. In our study, we assume that both the measured frequency range and the outer boundary of a target in a given MR image are known, which means the POCS method can be applied for improving the spatial resolution of the given MR image by representing the knowledge with two convex sets in an image space.

Super-resolution of images is an ill-posed problem, and regularized optimization techniques are widely used for solving the super-resolution problem. One of the most popular regularizers is total variation (TV) of images (e.g., [21–23]), which helps to reduce ringing artifacts and noises in images while preserving the edges [21, 24]. In addition to TV, rank regularization has also attracted much attention for solving ill-posed problems such as an image completion problem (e.g., [25–27]). It is reported that one can improve the performance of image super-resolution by combining the TV regularization with the low-rank one [23]: high-resolution images are obtained by minimizing a cost function, in which both a TV regularization term and a low-rank regularization term are included.

Recently, there are more and more deep learning-based super-resolution proposed. Learning-based approaches exploit internal or external database to super-resolve an image [28–31]. For example, SRGAN [32] trains and generates high-frequency patterns of input images. LAPGAN [33] exploits the Laplacian pyramid of images, where the high-resolution images can be well represented as straightforward hierarchy summations of generated high-frequency patterns and a low-resolution image. The resultant images of them are extremely realistic.

Learning-based methods, however, can largely improve spatial resolution of input images only when sufficient number and variation of training data are available and target images can be regarded as drawn from the probability distribution the training data represent. For example, given a set of sufficiently large number of training CT images of healthy subjects, one can improve the spatial resolution of a CT image of a new healthy subject well but it would be difficult to improve the resolution if it is a CT image of a subject with tumors. It should be noted that, in medical image processing, collecting sufficient number and variety of medical images for the training is challenging [34]. Compared with learning-based approaches, mathematical model-based approaches, which include POCS methods, can be applied to images that are consistent with the employed mathematical models and are not affected by the bias of the collected training data.

In this study, we propose a framework for incorporating the Gerchberg algorithm into a regularized optimization based method of super-resolution. In this framework, we can use the knowledge of the outer contour of a target and of the measured frequency range with the conventional regularizers simultaneously for computing higher spatial resolution images. Combining TV regularization with the Gerchberg term, one can suppress ringing artifacts often generated by the Gerchberg method. Here, it should be noted that the incorporation of the Gerchberg method into regularized optimization based methods is not so straightforward because the Gerchberg method obtains high-resolution images not by explicitly minimizing some cost function but by iteratively projecting an image onto convex sets. The main contributions of the present study are as follows: (1) we reformulate the projections in the Gerchberg super-resolution algorithm using linear matrix equations, (2) we formulate a convex optimization problem, in which the reformulated projections and the low-TV/low-rank regularization are represented in a cost function and constraints, (3) we explicitly describe the algorithm for solving the convex optimization problem with the alternating direction method of multipliers (ADMM), and (4) we present extensive experimental evaluations conducted using the proposed method.

The proposed method has the following theoretical limitations on the input MR images in order to solve an inverse problem: (i) the boundary of an image object can be labeled in a reasonable time and the backgrounds are composed of its blur and noise, (ii) the blur kernel or PSF of the observation is known in advance, and (iii) the image noise obeys the normal distribution.

The remainder of this paper is organized as follows. In Section 2.1, we explain the notations used in this study. Next, we provide a problem statement regarding the present study in Section 2.2. In Section 2.3, we review the Gerchberg algorithm and recent regularization-based approaches. The proposed method and the description of its explicit solvers are explained in Section 2.4. Variational experimental results are presented in Sections 3–3.5. In Section 3.6, we discuss the behavior and various aspects of the proposed method. Finally, we give our conclusions in Section 4.

## 2. Materials and Methods

### 2.1. Notations

In this study, a vector is denoted by a bold small letter **a** and a matrix is denoted by a bold capital letter **A**. A 3D tensor is denoted by a bold calligraphic letter *A*. The (*s*, *t*)-th entry of a matrix **A** is denoted by **A**_*st*_ and the (*s*, *t*, *u*)-th entry of a 3D tensor *A* is denoted by *A*_*stu*_.

Given a vector **a**, the tensor folding operator is denoted by fold(**a**) : **a** ∈ *ℝ*^*I*_1_*I*_2_*I*_3_×1^ → *A* ∈ *ℝ*^*I*_1_×*I*_2_×*I*_3_^, and its adjoint operator is vec(*A*) : *A* ∈ *ℝ*^*I*_1_×*I*_2_×*I*_3_^ → **a** ∈ *ℝ*^*I*_1_*I*_2_*I*_3_×1^. Given a vector **a**, its matricization is denoted by mat(**a**) : **a** ∈ *ℝ*^*IJ*×1^ → **A** ∈ *ℝ*^*I*×*J*^. Given a tensor *A*, the *i*-th mode unfolding operator is denoted by unfold_*i*_(*A*) : *A* ∈ *ℝ*^*I*_1_×*I*_2_×*I*_3_^ → **A** ∈ *ℝ*^*I*_*i*_×∏_*l*≠*i*_*I*_*l*_^, and its adjoint operator is fold_*i*_(**A**) : **A** ∈ *ℝ*^*I*_*i*_×∏_*l*≠*i*_*I*_*l*_^ → *A* ∈ *ℝ*^*I*_1_×*I*_2_×*I*_3_^.

Given that **A** = **U**Σ**V**^T^ is the singular value decomposition for a matrix **A**, a singular value soft-thresholding operator [25, 35] is defined as

(1) $$\begin{array}{c} SVT_{\tau }\left(\;A\;\right)=U\Sigma_{\tau }V^{T}, \end{array}$$

where Σ_*τ*_ = diag([max(*σ*_1_ − *τ*, 0), max(*σ*_2_ − *τ*, 0), ⋯, max(*σ*_*I*_ − *τ*, 0)]^T^) and *σ*_*ι*_ is the *ι*-th singular value of **A**. The operator ∘ is the Hadamard (element-wise) product.

### 2.2. Problem Statement

Without loss of generality, we can assume that a field of view (FOV) of an MR image is a cubic space. Let the side length of the cubic FOV be denoted by *L* and let the three mutually orthogonal directions corresponding to the sides of the cubic FOV be denoted by a *X*-axis, a *Y*-axis, and a *Z*-axis.

For simply describing the method, we assume that the slice thickness and the slice spacing are equal and that an MR image consists of *n* slice images, each of which has *N*_*c*_ × *N*_*c*_ voxels. It follows that the voxel size along the through-slice direction is given by *M* = *L*/*n* and that the voxel size in each slice image is given by *m* × *m*, where *m* = *L*/*N*_*c*_. *M* > *m* holds in many MR images in order to assure high SNR. (Increase of the slice thickness would degrade the spatial resolution along the through-slice direction, though the SNR of each slice image would be improved by the increase of the slice thickness because the quantity of hydrogen nuclei included in the measured slice increases and the magnitude of the signals emitted by the hydrogen nuclei also increases.) Let the scaling factor be denoted by *β*, where *β* = *M*/*m* = *N*_*c*_/*n*. The spatial resolution along the through-slice direction is *β* times lower than the resolution along the in-plane directions in an MR image.

In the experiment here, we assume that two MR images are given. When multiple MR images are given, it is assumed that the MR images are obtained with mutually orthogonal directions of slice-selective gradient. Let 3D tensors, *X*_1_ ∈ *ℝ*^*N*_*c*_×*N*_*c*_×*n*^, *X*_2_ ∈ *ℝ*^*N*_*c*_×*n*×*N*_*c*_^, denote MR images of the same FOV obtained with the slice-selective gradient parallel to the *Z*-axis and the *Y*-axis, respectively. Let a tensor *I* ∈ *ℝ*^*N*_*c*_×*N*_*c*_×*N*_*c*_^ denote an *N*_*c*_ × *N*_*c*_ × *N*_*c*_ isotropic noise-free MR image of the FOV obtained by an ideal MR scanner. It is assumed that any measured MR image of the FOV, *X*_*d*_, can be generated from *I* by appropriately eliminating higher frequency components in the corresponding direction of the slice-selective gradient followed by downsampling by *β* = *N*_*c*_/*n*.

**Figure 1:**
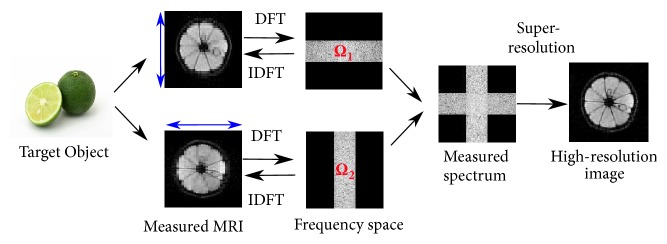
Flow of MR image acquisition and super-resolution. The specimen is measured anisotropically from different directions. The blue arrows denote the through-slice directions. MR images are obtained with a narrow bandwidth along the through-slice directions. In most MR images, the spatial resolution along the through-slice direction is more than three times lower than that in the other two directions. There are still unknown high-frequency components even when a number of measurements are available. These components are completed by super-resolution.

Let the Fourier transform of *X*_*d*_ be denoted by *F*_*d*_ and let *Ω*_*d*_ denote a frequency region only in which the Fourier components of *X*_*d*_ are measured: Outside of the region, ${\bar{\Omega }}_{d}$, in the frequency space, the frequency components are zero. As shown in Figure 1, it should be noted that *Ω*≔*Ω*_1_ ∪ *Ω*_2_ does not cover the whole spectrum space and that diagonal high-frequency regions are not observed in any of the images. The objective here is to estimate/complete the unknown frequency components and reconstruct a high-resolution MR image.

It should be noted that there is a fundamental difference between the assumptions for our input image and that for compressed sensing MRI. For compressed sensing, it is assumed that the input is random from a sampled k-space, which includes both high- and low-frequency components in an incoherent manner [36–38]. By contrast, our input MR images have been taken already and only the low-frequency components are given; thus, the completion approaches used for compressed sensing cannot be applied.

### 2.3. Existing Methods for Super-Resolution

#### 2.3.1. POCS Algorithm

POCS is one of the frameworks for the super-resolution [8, 15]. There are various constraints which the ground truth image must satisfy. In the image domain, some of those constraints are expressed as forms of convex sets where the reconstructed image must be included. A POCS algorithm projects an input image onto the convex sets one by one repeatedly to obtain the unique solution. The convex sets, which we refer to also as* models*, vary depending on the various conventional POCS methods. For example, there are methods which employ data fidelity and nonnegativity as the models [15, 16]. We focus on the Gerchberg algorithm, which is one of the earliest POCS algorithms. The Gerchberg algorithm employs two models: the fidelity of the spectrum and the boundary of the region where the object exists.

**Figure 2:**
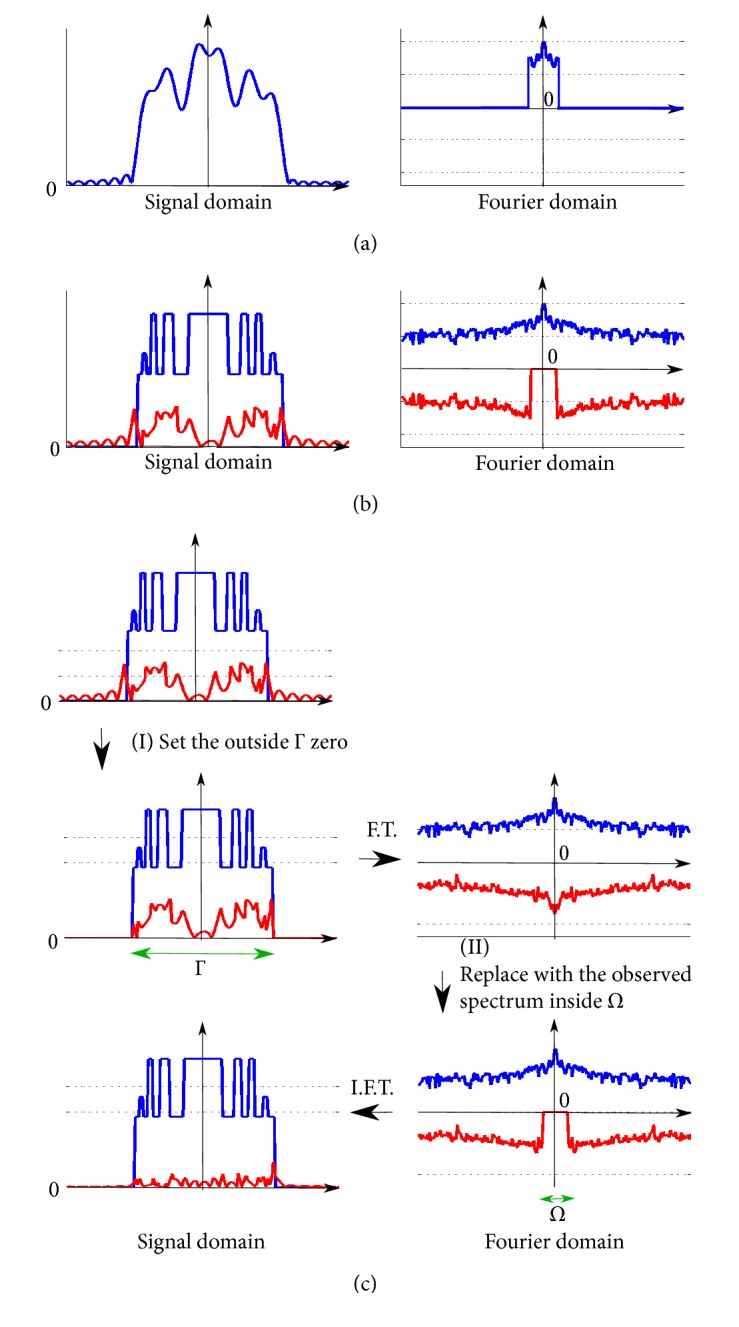
Illustration of the Gerchberg algorithm. (a) The observed image (left) and its spectrum (right). (b) The interpretation of (a). The observed image is the sum of the true signal/spectrum (blue line) and the error signal/spectrum (red line). (c) The procedure followed by the algorithm. The error spectrum is reduced by iterating in both the signal and Fourier domains.

In the remainder of this section, we introduce the Gerchberg algorithm [7, 39]. The Gerchberg algorithm assumes that an image signal is spatially finite and that the outer boundary of the finite region, Γ, is known in advance. In the Gerchberg algorithm, an image is super-resolved by iteratively repeating two projections onto two convex sets: (I) setting the image signal outside of Γ, denoted by $\bar{\Gamma }$, to zero; (II) updating the spectrum within the observed region, *Ω*, so as to remain as the observed value. An example of the algorithm in the case of a one-dimensional signal is shown in Figure 2. The Gerchberg algorithm is summarized in Algorithm 1.

Let *X* ∈ *ℝ*^*N*_1_×*N*_2_×*N*_3_^ denote an image signal and let *F* denote its Fourier transform. In the initial state, *F* = *F*_0_, where *F*_0_ denotes the observed spectrum. Let *P*_Γ_ ∈ {0,1}^*N*_1_×*N*_2_×*N*_3_^ be a 3D binary label array such that 0 and 1 indicate the outside and inside voxels of the target object, Γ, respectively. The first step of the algorithm is given by *X* ← *P*_Γ_∘IDFT(*F*), where IDFT(·) denotes a linear operator that provides the inverse 3D discrete Fourier transform (DFT). This operation sets the image signal outside Γ (inside $\bar{\Gamma }$) to zero. Let *P*_*Ω*_ denote a 3D index array such that 0 and 1 indicate $\bar{\Omega }$ and *Ω*, respectively. The second step of the algorithm is then given by $F\leftarrow P_{\Omega }\circ F_{0}+P_{\bar{\Omega }}\circ DFT(X)$, where DFT(·) is a linear operator that provides the 3D-DFT. This operation replaces the calculated spectrum, *F*, in the region *Ω* with the observed spectrum, *F*_0_. These two steps are repeatedly conducted and the resultant *F* will converge to the unique solution.

The converged unique solution should be the true spectrum under the ideal conditions. The observed spectrum *F*_0_ is interpreted as being the sum of two types of spectra: the true spectrum to be restored and the* error spectrum* that represents the difference between the true spectrum and the observed spectrum (Figures 2(a) and 2(b)). It should be noted that IDFT(*F*_0_) denotes a low-resolution image that is blurred because the high-frequency components are not observed. The blurred image is interpreted as being the sum of the true high-resolution image to be restored and the* error image* that is the IDFT of the error spectrum, as shown in Figure 2. In step (I), the operator *P*_Γ_ reduces only the power of the error image by removing the blur image components in $\bar{\Gamma }$. In step (II), the operator *P*_Γ_ has no effect on the true signal, which is zero in $P_{\bar{\Gamma }}$. Here, one can remove only the energy of the error spectrum by replacing only the spectrum components within *Ω* with the observed values, *F*_0_, because the true spectrum is observed in the lower frequency region, *Ω*. Repeating the two projections (I) and (II) described above, the error spectrum converges toward zero and the resulting spectrum converges toward the true spectrum.

In practice, however, it is assumed in the Gerchberg algorithm that the observed low-frequency spectrum is strictly the same as that of the ground truth image. Thus the resultant image reaches an invalid solution that deviates from the true spectrum when the observed image is contaminated with some noise. It is also assumed that the object exists in inner Γ in the image domain. This assumption means that Γ should not invade the true region where the object of ground truth exists. On the other hand, if Γ is redundant from the true region, the reconstruction performance would more or less degrade despite the algorithm attempt to reach the ground truth. It is also known that the reconstructed image could be contaminated by ringing artifacts even under the ideal conditions [9–11]. In order to improve the performance, we introduce regularization approaches. In the next subsection, we describe the introduced regularizers.

#### 2.3.2. Regularization-Based Methods

In this section, we review conventional regularization-based super-resolution approaches introduced in our method: TV regularization, rank regularization, and their combination. TV is an evaluation measure for the smoothness of an image and its minimization plays an important role in solving the inverse problem in signal processing, such as denoising, interpolation, deconvolution, and super-resolution [23, 24, 40–43]. Using simple notations, super-resolution problem with TV regularization can be formulated as

(2) minimizeX DSX0,X+λXTV,

where *X*_0_ is the observed signal, ‖·‖_TV_ is the total variation, and *D*_S_(·), which is some kind of distance measure between *X*_0_ and *X*, evaluates the image fidelity. TV is defined as

(3) $$\begin{array}{c} {\left\|\;X\;\right\|}_{TV}≔\underset{s,t,u}{\sum }\sqrt{\overset{3}{\underset{d=1}{\sum }}{\left[\nabla_{d}\left(X\right)\circ \nabla_{d}\left(X\right)\right]}_{stu}}, \end{array}$$

where *s*, *t*, *u* are voxel indices for an 3D tensor and ∇_*d*_ is a partial differential operator with respect to the *d*-th axis of a 3D image.

In many cases, *D*_S_(·) is a linear operator such as *L*_2_-norm for the image fidelity considering the existence of Gaussian noise. Thus the problem in (2) is often a convex optimization problem. However, classical gradient-based and Newton-like methods cannot be used since ‖*X*‖_TV_ is not a differentiable function. The primal-dual splitting (PDS) method [41, 43, 44], ADMM [45, 46], and the majorization-minimization (MM) algorithm [47] can solve the TV regularization problem in an efficient manner.

For the regularization term, it is also possible to use the low-rank property in the image restoration. For tensor completion, regularization with rank is known to obtain superior reconstructions [26]. The rank of a matrix is not a convex function, but its approximation can be minimized as convex optimization using the trace norm [48–50]. The trace norm of a matrix is defined as the sum of all the singular values. The rank of a tensor can also be approximated effectively as the trace norm of a tensor, which is defined as the weighted sum of all the matrix trace norms for the individual mode-matricization of a tensor [25]:

(4) $$\begin{array}{c} {\left\|\;X\;\right\|}_{*}=\overset{N}{\underset{i=1}{\sum }}\alpha_{i}{\left\|\;X_{\left(i\right)}\;\right\|}_{*}, \end{array}$$

where *𝒩* is the number of tensor dimensions and {*α*_*i*_}_*i*=1_^*𝒩*^ are parameters that satisfy ∑_*i*=1_^*N*^*α*_*i*_ = 1 and *α*_*i*_>. **X**_(*i*)_ ∈ *ℝ*^*N*_*i*_×*N*_*j*_*N*_*k*_^, where *i*, *j*, *k* ∈ {1,2, 3}  (*i* ≠ *j* ≠ *k*) is the matrix obtained by **X**_(*i*)_ = unfold_*i*_(*X*). In the following, *α*_*i*_ = 1/*𝒩* and *𝒩* = 3 are set because 3D MR images are 3D tensors. Then, the 3D tensor completion problem regularized by rank is configured as

(5) minimizeX DSX0,X+λX∗,

where *P*_Ψ_ ∈ {0,1}^*N*_1_×*N*_2_×*N*_3_^ indicate the indices where the elements are observed. The problem in (5) can be optimized by ADMM using the singular value thresholding operator (1) [25].

In an application of regularized-based super-resolution for MR imaging, [23] also imposed rank regularization on problem (2) and achieved a satisfactory improvement in performance. They configured the optimization problem as

(6) minimizeX DSX0,X+λTVXTV+λLRX∗.

In practice, the tensor trace norm can be minimized by using slack variables for each dimension [23, 25].

### 2.4. Proposed Method

We have introduced two types of super-resolution methods: the Gerchberg algorithm [7, 39] and regularization-based approaches [21–23, 25]. The Gerchberg algorithm can be characterized by the global boundary prior and the observed spectrum maintenance. By contrast, regularization-based methods can be characterized as performing super-resolution by signal fitting with a local smoothness (low-TV) or global similarity (low-rank) prior, which is generally satisfied in natural images. The proposed super-resolution algorithm combines both strategies and modifies them by including signal and spectral fitting with smoothness (low-TV) and global (low-rank and the boundary) priors.

#### 2.4.1. Outline of the Proposed Method

The proposed method is obtained by combining LRTV super-resolution [23] and the Gerchberg algorithm. As mentioned in Section 2.3.1, the Gerchberg algorithm is given in the form of an iterative projection with *P*_Γ_, *P*_*Ω*_, and $P_{\bar{\Omega }}$, and hence these two methods cannot be combined straightforwardly. Thus, in order to impose regularization technique on the Gerchberg algorithm, we first give a reinterpretation of the Gerchberg algorithm. The Gerchberg algorithm can be reinterpreted as solving the following convex optimization problem for the spectrum *F*:

(7) minimizeF PΩ∘F0−FF2,+iΓX,s.t. X=IDFTF

where *F*_0_ is the observed spectrum (see Section 2.4 for details). In problem (7), *i*_Γ_(*X*) is the following indicator function:

(8) $$\begin{array}{c} i_{\Gamma }\left(\;X\;\right)=\left\{\begin{array}{cc} 0 & \text{if}\;\;P_{\bar{\Gamma }}\circ X=O\\ \infty & otherwise \end{array}\right. \end{array}$$

where $P_{\bar{\Gamma }}=1-P_{\Gamma }$ and *O* ∈ {0}^*N*_1_×*N*_2_×*N*_3_^ is a zero 3D array. The first term in problem (7) represents fitting the spectrum *F* with *F*_0_ for the pass-band, considering the existence of Gaussian noise with the observation. The second term, *i*_Γ_(*X*), implies that all the outside voxels of the image are zero. Each linear term in (7) corresponds to the projection onto the convex set in the signal or Fourier domains in the Gerchberg algorithm.

Based on (7) and LRTV regularization, we propose to solve the following convex optimization problem:

(9) minimizeF λTVXTV+λLRX∗+iΓX+12PΩ∘F0−FF2,s.t. X=IDFTF,

where *λ*_TV_, *λ*_LR_ > 0 are parameters that control the balance between the respective terms. In contrast to the image-fidelity-based problems in (2) and (6), the error terms in the proposed method are for fitting the Fourier spectrum. The image/frequency fidelities are regularized/constrained by TV, rank, and the region Γ. The behavior of each term in (9) is considered in Sections 3.1 and 3.6.

The following notice must be considered before the optimization of problem (9). First, the Gerchberg algorithm assumes that the spectrum profile is a rectangular function. However, in clinical MR imaging, the spectrum of the slice profile forms a Gaussian or windowed-sinc function (e.g., [51–53]). With this notice, we use *F*_0_′ = *F*_0_⊘*P*_Ξ_ instead of *F*_0_, where *P*_Ξ_ is the spectrum of the slice profile along through-slice directions and ⊘ is the element-wise division operator. Next, a slack variable is used for each dimension, *M*_*i*_ ∈ *ℝ*^*N*_1_×*N*_2_×*N*_3_^, for the optimization process. Considering (4) and setting *i*_Γ_(*X*) into the constraints, (9) is rewritten as

(10) minimizeF λTVXTV+λLRN∑i=1NMii∗+12F0′−PΩ∘FF2,s.t. X=IDFTF, O=PΓ¯∘X, Xi=Mii,i=1,…,N

where **M**_*i*(*i*)_ = unfold_*i*_(*M*_*i*_). The constraints can be simplified by introducing a variable **V**_*i*_ with respect to the third constraint. Then, the vector form of the proposed problem (10) with relaxation is given by

(11) minimizef λTVxTV+λLRN∑i=1NMii∗+12f0′−RΩf22+ϵ2∑i=1Nx−mi+vi22−vi22,s.t. x=Gf, 0=RΓ¯x,

where **x** = vec(*X*), **x**_0_ = vec(*X*_0_), **f** = vec(*F*), **f**_0_′ = vec(*F*_0_′), **R**_*Ω*_ = diag(vec(*P*_*Ω*_)), **m**_*i*_ = vec(*M*_*i*_), $R_{\bar{\Gamma }}=diag(vec(P_{\bar{\Gamma }}))$, and 0 = vec(*O*). **G** is a linear operator (matrix) that gives the inverse 3D DFT. *ϵ* > 0 is an additional parameter for the fitting term of the slack variables. Note that all of the terms and constraints in (11) are convex or linear; thus, (11) is a convex optimization problem that can be solved using the PDS, ADMM, and MM algorithms.

#### 2.4.2. An Optimization Algorithm

Several algorithms can be used to solve (11). In this section, we introduce a convex optimization algorithm that uses ADMM [45] as an example.

To hold proximity, we reformulate (11) as

(12) minimizef λTVY1,2+λLRN∑i=1NMii∗+12f0′−RΩf22+ϵ2∑i=1Nx−mi+vi22−vi22,s.t. Y=L1x,L2x,L3x, x=Gf, 0=RΓ¯x,

where ‖[**z**_1_, **z**_2_,…,**z**_*N*_]^T^‖_1,2_≔∑_*n*=1_^*N*^‖**z**_*n*_‖_2_ is an *l*_1,2_-norm and **L**_*d*_ is a partial differential operator with respect to the *d*-th axis. The first constraint can be rewritten as **y**≔vec(**Y**) = [**L**_1_^*T*^, **L**_2_^*T*^, **L**_3_^*T*^]^*T*^**x**≕**Lx**.

The augmented Lagrangian of (12) is given by

(13) Lf,x,y,z,α,γ=λTVY1,2+λLRN∑i=1NMii∗+12f0′−RΩf2+ϵ2∑i=1Nx−mi+vi22−vi22+z,y−Lx+α,x−Gf+γ,RΓ¯x+ρ2y−Lx22+ρ2x−Gf22+ρ2RΓ¯x22,

where **z**, **α**, and **γ** are the Lagrange coefficients and *ρ* > 0 is the penalty weight. By minimizing (13) with respect to **f**, **x**, **y**, and **m**_*j*_, the following update rules can be obtained:

(14) fk+1=RΩ+ρI−1RΩf0′+GTαk+ρxk,

(15) xk+1=ρI+RΓ¯+LTL+NϵI−1ϵ∑i=1Nmi−vi−LTzk+α+RΓ¯γ+ρGf−LTyk,

(16) Yk+1st=max⁡1−λTV·ρwsk2−1,0wstk,

(17) mik+1=vecMik+1=vecfoldiSVTλLR/NϵunfoldjXk+1+Vik,

(18) vik+1=vik+x−mi

where **I** is an identity matrix, **w**_*s*_^*k*^ = [*w*_*s*1_^*k*^, *w*_*s*2_^*k*^, *w*_*s*3_^*k*^]^*T*^, and *w*_*st*_^*k*^ = [mat(**Lx**^*k*+1^ − *ρ*^−1^**z**^*k*^)]_*st*_. The derivations of the update rules given above are described in the Appendix. The Lagrange multipliers are updated by

(19) zk+1=zk+ρyk+1−Lxk+1,

(20) αk+1=αk+ρxk+1−Gfk+1,

(21) γk+1=γk+ρRΓ¯xk+1.

For (15), the conjugate gradient method can be used instead of the inverse matrix, which requires a large amount of calculations. The parameters are updated by repeatedly applying (14)–(21) alternatively until the convergence of the original cost function in (11). The proposed method with the above notations is summarized in Algorithm 2.

## 3. Results and Discussion

We examined the characteristics of the proposed method using MR images of a brain phantom and of human head portions. The experiments were performed with brain phantom images [54] and with clinical MR images.

For the phantom images, different four images, which vary together in the modality (T1 or T2 weighted) and in the pathological status (with or without lesion), were used. Each phantom image had a spatial resolution of 1 × 1 × 1mm^3^. After setting an original phantom image as the ground truth, we simulated two anisotropic observed images by downsampling toward different orthogonal directions. The blurring kernel for downsampling was rectangular (average) toward the downsampling direction and we assumed that the slice profile in the signal domain was rectangular along the through-slice direction. Thus, the two observed images had spatial resolutions of 1/*β* × 1 × 1mm^3^ and 1 × 1/*β* × 1mm^3^, where *β* is the scaling factor. The variational settings of scaling factors and noise levels are simulated for the observations. 16 settings of the observations in total, which vary together in modalities, in pathological status, in scaling factors, and in noise levels, were simulated and are shown in Table 1.

As for the clinical images, 37 MR images from the OASIS [55] were used for the experiment. MR images of different subjects were randomly chosen from* disc1* in OASIS-1 dataset. For each session (subject) of OASIS, the first scanned image was chosen for the evaluation. Each image had a spatial resolution of 1 × 1 × 1.25mm^3^. We employed the same observation procedure described above.

Using the MR images of a brain phantom, we at first examined the sensitivity of the accuracy of the image reconstruction against the hyperparameters in Section 3.1 and the computational time in Section 3.2. Then we compared the accuracy of the images reconstructed by the proposed methods and other conventional super-resolution methods and evaluated the reconstruction stability against the change of the noise level and scaling factor in Sections 3.3 and 3.4. As described, our method requires labeling the outer boundary of the target in a given image. We also evaluated the sensitivity of the reconstruction accuracy with respect to the accuracy of the labeled outer boundary in Section 3.4. Each performance was evaluated based on the peak signal-to-noise ratio (PSNR) in the target region of the restored images.

The performance of the proposed method is compared with the following existing methods: nearest neighbor interpolation (NN), bicubic interpolation, zero-padding in the Fourier space (ZP) [56], the Gerchberg algorithm [7], TV regularized super-resolution [22], and LRTV [23]. In the remainder of this paper, the proposed method is denoted as LRTVG and the proposed method without the rank regularization term (*λ*_LR_ = *ϵ* = 0) is denoted as TVG.

### 3.1. Sensitivity with respect to Hyperparameters

**Figure 3:**
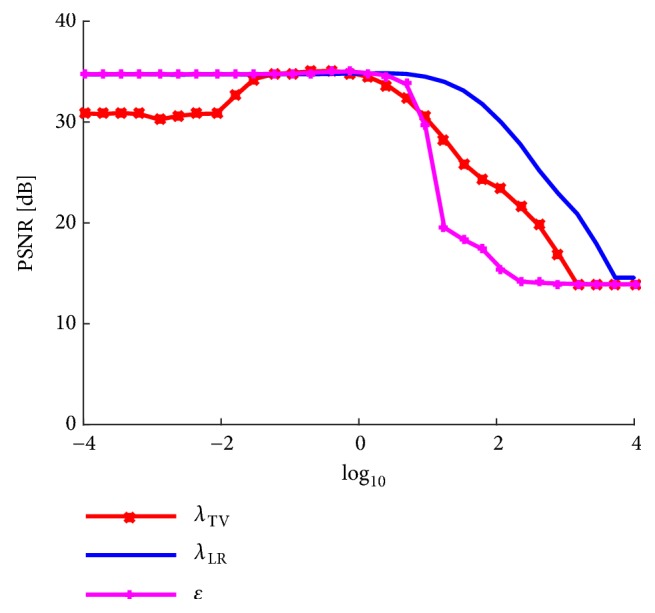
An example of PSNR results obtained using the proposed method in terms of the parameters *λ*_TV_, *λ*_LR_, *and*  *ϵ*. The input image was image (a) in Table 1.

First, we show behaviors of the parameters in the proposed model.  Figure 3 shows an example of changes in the PSNR with respect to *λ*_TV_, *λ*_LR_, and *ϵ* in (11). The changes in the PSNR with respect to *λ*_TV_ are more steeper than those with respect to *λ*_LR_. We can say that TV regularization must be more carefully tuned than LR regularization. *ϵ* should be set small enough so that the data fidelity term is retained well, as it is shown in Figure 3 that larger *ϵ* rapidly degrades the performance.

**Figure 4:**
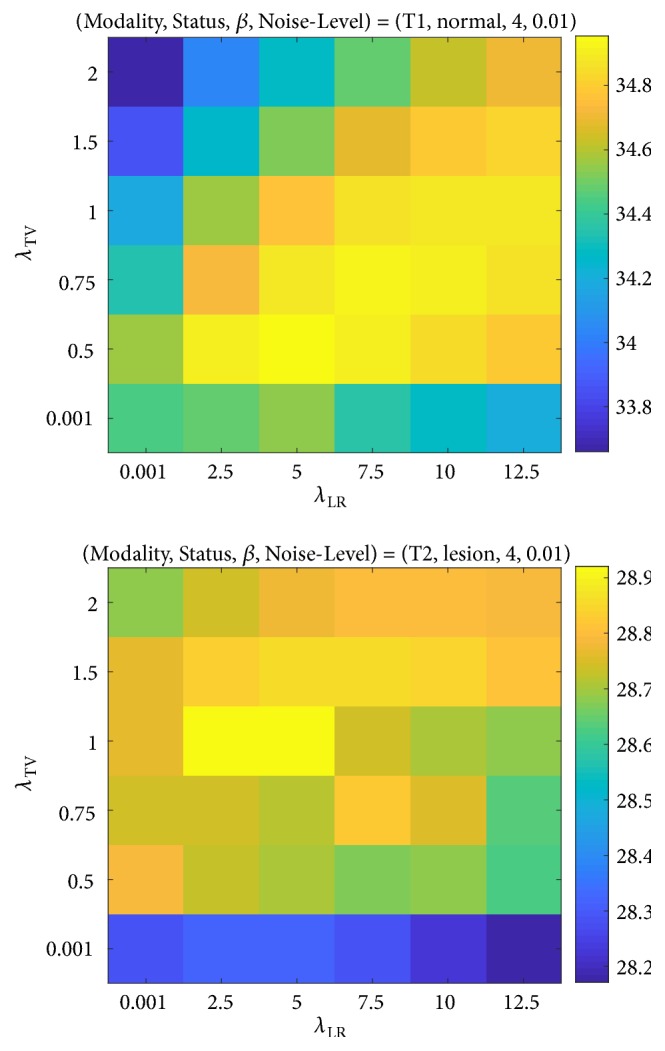
Examples of PSNR results of the proposed method when *λ*_TV_ and *λ*_LR_ are simultaneously changed. The input images were images (a) and (m) in Table 1.

The proposed method as well as TV and LRTV needs to tune the hyperparameters by the input image.  Figure 4 shows two examples of the different input images when *λ*_TV_ and *λ*_LR_ are simultaneously changed. As shown in Figure 4, *λ*_TV_ and *λ*_LR_, which actually control the regularization weights, should be varied by the image so as to exert the best performance for each image. The parametric behaviors for all the phantom images in Table 1 are also shown in Supplementary Material S7. Although the balance of the two parameters is case by case, it can be said at least that *λ*_TV_ and *λ*_LR_ would be larger when the noise level gets higher and that *λ*_TV_ and *λ*_LR_ would be smaller when the scale factor gets larger.

With those considerations, *λ*_TV_ and *λ*_LR_ were manually tuned by the input image while fixing *ϵ* = 0.01 in the following experiments.

### 3.2. Computational Time

**Figure 5:**
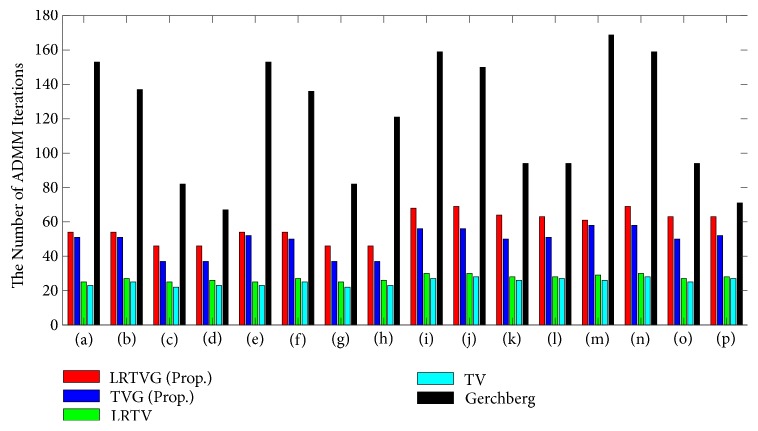
The numbers of iterations until the ADMM convergence. Each threshold of the stopping criteria was 1.0 × 10^−7^.

Next, we show the number of iterations each method took until the convergence in Figure 5, and the average processing times for one iteration in Table 2. The number of iterations until the convergence of the proposed method was larger than that of LRTV and TV. We suppose that this is because the additional two Lagrange multipliers, **α** and **γ**, are necessary for the optimization. The Gerchberg model, which also needs **α** and **γ** when solved with ADMM, took much more iterations than the other methods. It can be said that regularization accelerates the convergence speeds of the POCS methods.

Theoretically speaking, the computational orders of LRTVG/TVG for each iteration are equal to those of LRTV/TV. The experimental results in Table 2 show that computational times of LRTVG/TVG are a little longer than those of LRTV/TV, which is because several times of FFT are necessary for the proposed method compared with LRTV/TV. We discuss the computational complexity in detail in Section 3.6.

### 3.3. Comparison of Accuracy of Reconstruction

**Figure 6:**
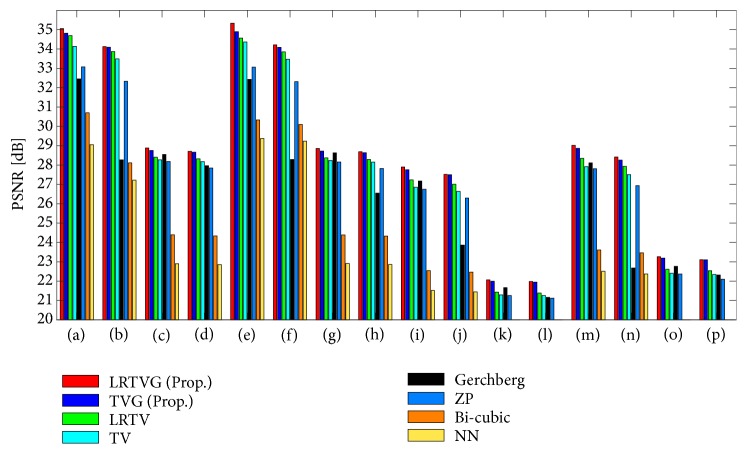
PSNR results of the variational simulations in Table 1.

**Figure 7:**
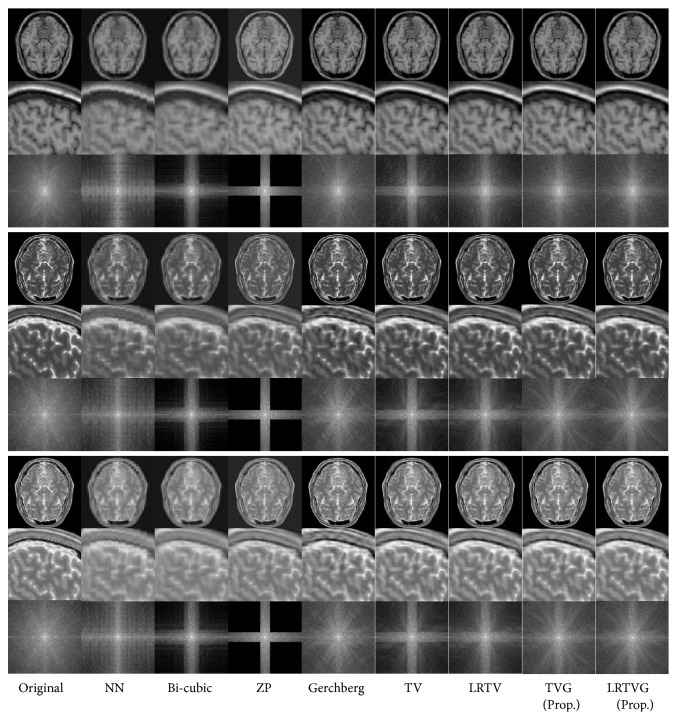
Reconstructed images of the brain phantom. Top three rows show illustrations of axial cross-sections, zoomed sagittal cross-sections, and the Fourier spectra of the T1-weighted brain without lesion (data (c)). The next three rows show those of the T2-weighted brain without lesion (data (k)), and the bottom rows show those of the T2-weighted brain with lesion (data (o)). *β* = 8 and the noise level was 1% for the observation settings.

In this section, we show the reconstruction accuracy of the proposed method compared to the existing methods: NN, bicubic interpolation, ZP, the Gerchberg algorithm, TV regularized super-resolution, and LRTV super-resolution. The restored images and PSNR results of the simulational observations in Table 1 are shown in Figures 6 and 7. All of the PSNR results were calculated in the region Γ. The parameters of TV, LRTV, and the proposed method are set by manual tuning as mentioned in Section 3.1

The simple interpolation methods, i.e., NN, bicubic, and ZP, generated blurred images. The Gerchberg algorithm was affected severely by ringing artifacts and noises, although sharp edges and high-frequency components can be observed in the results. Although the TV-based approaches preserved their edges clearly, the results of TV and LRTV lack high-frequency components in the Fourier space. The proposed method restored the high-frequency components as well as clear edges and had the best performance for all the input images in Table 1. All the PSNR results of T2-weighted images are clearly degraded compared to T1-weighted images. This would be because the image gradients in T2-weighted images more steeply change than those of T1 weighted images because of their modality characteristics.

**Figure 8:**
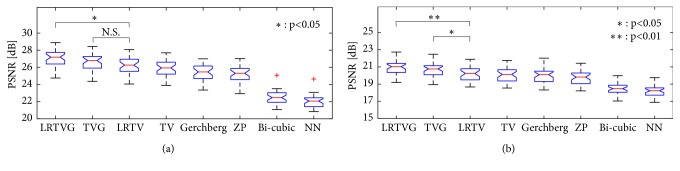
Box-plots of the PSNR results obtained using different methods with 37 images from OASIS. The proposed method (LRTVG and TVG) performed better than the other methods. (a) *β* = 6, (b) *β* = 12.

We also show the reconstruction accuracy of the 37 subjects from OASIS [55]. Box-plots of the results when *β* = 6 and when *β* = 12 are shown in Figure 8. The proposed method performed better than the others in terms of the PSNR. We examined the statistical significance of the performance difference of the proposed methods (LRTVG and TVG) and others. In case *β* = 6, the proposed LRTVG significantly outperformed all the other methods according to the t-test. In case *β* = 12, both of LRTVG and TVG significantly outperformed all the other methods.

### 3.4. Stability against Noise Level and Scaling Factor

**Figure 9:**
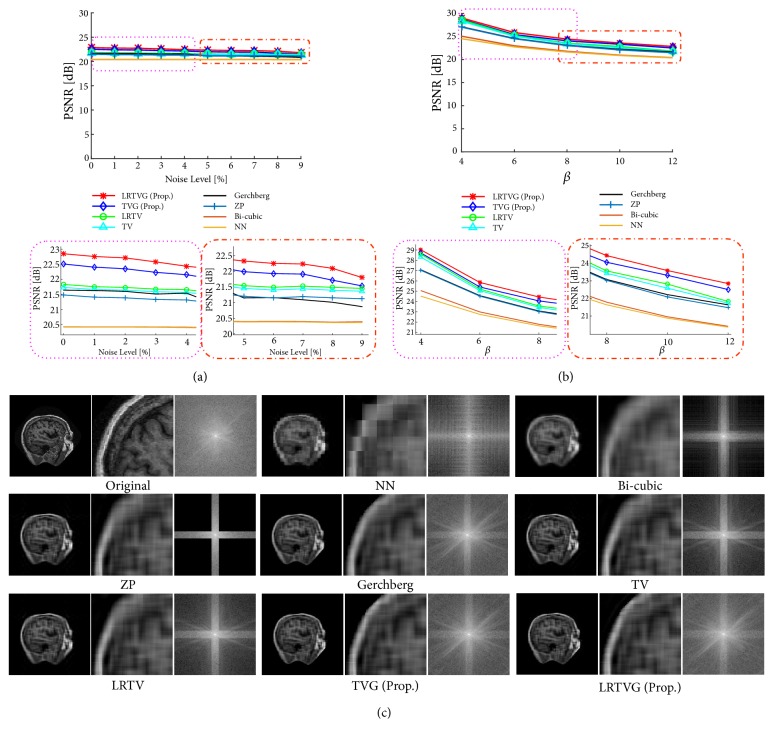
Experimental results obtained for an image from OASIS. (a) PSNR in terms of the Gaussian noise level (upper) and enlarged views (lower). (b) PSNR in terms of the scaling factor *β* (upper) and enlarged views (lower). (c) Reconstructed images and spectra obtained with *β* = 12.

We evaluated the change of the performance with respect to (i) noise level and (ii) scale factor.  Figure 9 demonstrates some examples of the experimental results.  Figure 9(a) shows PSNR for all noise levels when *β* = 12.  Figure 9(b) shows PSNR for all scaling factors when the observations are free of noise. The proposed method outperformed the other methods in all cases. With high noise level, the performance of the proposed method converges next to that of LRTV/TV. With the larger scaling factor, the proposed method performed significantly better compared with the other methods. These behaviors of the proposed method can also be observed in the results in Section 3.1, where the larger regularization weights work with high noise levels, and the smaller regularization weights work with large scaling factors.

### 3.5. Stability against Boundary Label

**Figure 10:**
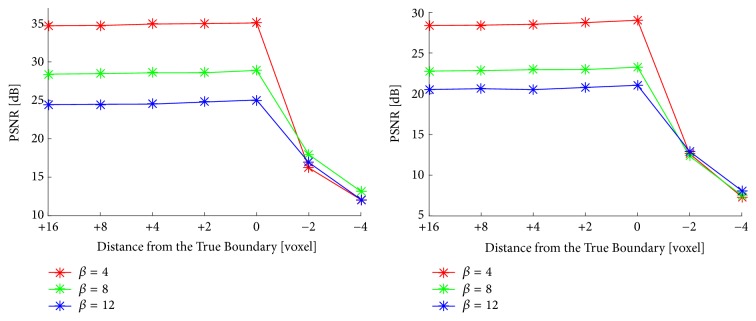
Differences in PSNR performance of the proposed method when the boundary became redundant (positive values) or insufficient (negative values) compared with the true boundary (the value is zero). The input image of the left figure was T1-weighted image of normal brain phantom, and that of the right one was T2-weighted image of lesional brain phantom.

Finally, we focus on the boundary constraint of the proposed method. The boundary contour will differ depending on the boundary detection procedure (e.g., manual, simple thresholding, or contour detection methods), so we examined the performance with respect to the boundary by dilating/shrinking the true boundary. The true boundary was dilated/shrunk by thresholding the distance map created from the level-set function.  Figure 10 shows the PSNR results as the distance from the true boundary changed. This distance corresponds to the difference in radius between the true boundary and the referred boundary. When the distance was positive (the referred boundary was redundant), the performance increased slightly toward a distance of zero where the boundary was perfectly accurate. The performance decreased steeply when the distance gets negative (the referred boundary was insufficient). This is obviously because not only the background but also the true signal is regarded as noise and the constraint is broken. Thus the target region must not be underestimated so that the proposed method works. When the boundary was more accurate, the proposed method performed better, but we need to be careful when setting the boundary not to encroach into the true target region.

### 3.6. Discussion

We consider the aspects of the proposed method and its other features. In terms of POCS approaches [8, 13, 15], the proposed method can be regarded as handling two convex sets: fidelity of the spectrum and signal boundary. In the proposed method, the projections onto these convex sets can be controlled by TV and rank regularization.

Actually, general regularization-based super-resolution methods simply assume that the resultant image will be smoother or spatially more similar than a noisy input image; there is no assumption for the restoration of fine structures themselves. On the other hand, POCS approaches can retrieve fine structures theoretically as described in Section 2.3.1. However, POCS approaches strictly obey their convex sets, and sometimes they will not be able to compete with images which are out of their model. For example, the Gerchberg model cannot compete with noisy inputs theoretically and with ringing artifacts, which is caused by the discrete Fourier transform. Unlike those methods, the proposed method can allow both the restoration of fine structures and the existence of noise or artifacts. Fundamental behaviors of those methods can also be observed in Supplementary Materials S1-S5.

The same as the general regularization-based super-resolution methods, influence of noise is controlled by regularization terms. The weights for regularization are decided by *λ*_TV_ and *λ*_LR_. As *λ*_TV_ becomes larger, image gradients will get sparse and the restored image becomes smoother. When *λ*_LR_ becomes larger, the reconstructed image becomes low rank. Results in Section 3.1 showed that the behaviors of the two parameters vary by the image. Note that the super-resolution model of the proposed method actually includes the Gerchberg model and equals it when *λ*_TV_ = *λ*_LR_ = 0 and *P*_Ξ_ denotes rectangular profile.

In addition to the regional constraint limitation described in Section 3.5, there are some implicit limitations of the proposed method considered: (i) the PSF is known in advance (the problem to be solved is not the blind deconvolution), (ii) the outer boundary of the target can be labeled in a reasonable time, and (iii) image noises can be well represented by the normal distribution. The limitations (i) and (iii) are derived from the data fidelity term of the proposed model. TV and LRTV also have limitations (i) and (iii). Without (i), when the PSF is unknown, the problem to be solved is the nonconvex optimization and cannot be solved easily. When the image noises do not obey the normal distribution, the error function must be corrected by the noise distribution, or some preprocessing is necessary for denoising. Limitation (ii) would be necessary for the clinical applications and would rarely be broken. When limitation (ii) is broken, the observed image would be contaminated severely with noises, and the target region/background cannot be determined easily. However, in the case when the super-resolution is necessary for an input MR image, the slice thickness is large enough and the noise level of the observed image would be small enough so that the labeling could be easily conducted.

We also discuss the computational complexity of the proposed method. In the followings, a tensor of the size *N*_*c*_ × *N*_*c*_ × *N*_*c*_ is assumed, and the total number of the voxels is *N*_*c*_^3^. First of all, (16) and (18)–(21) cost *O*(*N*_*c*_^3^) obviously. Equation (14) costs *O*(*N*_*c*_^3^log⁡*N*_*c*_) for the FFT when it is calculated by each fiber of a 3D tensor. When the multiplication of the convolution-matrix and its inversion in (15) are calculated in the Fourier space, the cost of the inverse multiplication itself can be reduced to *O*(*N*_*c*_^3^). Thus (15) also costs only *O*(*N*_*c*_^3^log⁡*N*_*c*_) for the FFT. For (17), the size of a matrix, unfold_*j*_(**X**^*k*+1^ + **V**_*i*_^*k*^), is *N*_*c*_ × *N*_*c*_^2^ and its SVD costs *O*(*N*_*c*_^4^). Therefore *O*(*N*_*c*_^4^) for the proximal operator of rank is the worst computational cost for each iteration, which is the same as that of LRTV.

The convergence speed/number of iterations of the proposed method are slower/larger than that of LRTV. As mentioned in Section 3.2, this would be because the additional two Lagrange multipliers, **α** and **γ**, are necessary for the optimization. The convergence speed also depends on the optimization frameworks and total variation minimization algorithms employed.

There are several future works considered in this study. We proposed the super-resolution model itself and the parameters of the proposed method are hand-tuned so far as discussed above. Actually, there are several methods proposed for automatically tuning the parameters of the TV optimization (e.g., [57]), and the proposed model also would be able to apply these autotuning methods. For the clinical application, this would be necessary in order to process in shorter time. In order to achieve more accurate results, processing the segmentation of the target region and the super-resolution of the image at the same time can be considered. This can be performed by optimizing **X**, **F**, and $P_{\bar{\Gamma }}$ at the same time. The fact that the fidelity term would explode when the target region is underestimated could be exploited for the simultaneous optimization. However this would lead the model to be an nonconvex optimization problem, which is much more difficult for initialization and for the selection of the solvers. The other work to enforce the performance is introducing the regularization based on deep neural networks like [58, 59], for example. Reference [58] uses trained DCNN and its population as the regularization of the signal fidelity term. Reference [59] exploits the structure itself of deep neural networks for the regularization. The combination of POCS optimization and deep neural networks would lead to higher performance.

## 4. Conclusions

In this study, we proposed a new super-resolution model where the Gerchberg algorithm is regularized by rank and TV. In order to configure the optimization problem, we first reformulated the Gerchberg algorithm as a convex optimization problem. We applied our method to MR images in order to obtain high resolution with a high SNR by using anisotropic measurements from the axial, coronal, or sagittal directions. The experimental results showed that our super-resolution technique dramatically reduced noise and ringing caused by the Gerchberg method and it also performed better than LRTV super-resolution and the other methods considered.

## Figures and Tables

**Algorithm 1:**
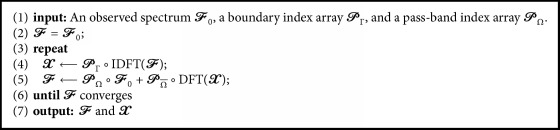
Gerchberg algorithm [7, 39].

**Algorithm 2:**
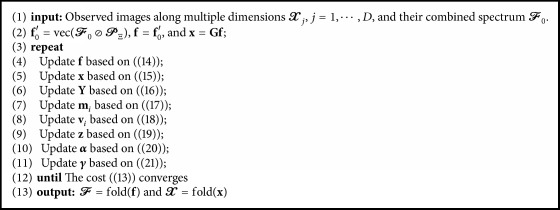
LRTVG algorithm.

**Table 1:** Correspondences of simulational settings and image indices. N/L of the **Status** row means the normal/lesional brain. Data ID is used to index in Figures 6-7 and in Supplementary Material S7.

**Data ID **	(a)	(b)	(c)	(d)	(e)	(f)	(g)	(h)	(i)	(j)	(k)	(l)	(m)	(n)	(o)	(p)
**Modality **	T1	T1	T1	T1	T1	T1	T1	T1	T2	T2	T2	T2	T2	T2	T2	T2
**Status **	N	N	N	N	L	L	L	L	N	N	N	N	L	L	L	L
*β*	4	4	8	8	4	4	8	8	4	4	8	8	4	4	8	8
**Noise level **[%]	1	5	1	5	1	5	1	5	1	5	1	5	1	5	1	5

**Table 2:** The average processing times for one iteration. The image size was 220 × 220 × 220.

**Methods **	LRTVG	TVG	LRTV	TV	Gerchberg
**Avg. Process. Time (sec.) **	5.73	4.81	5.24	4.54	4.86

## Data Availability

MR phantom images used to support findings of this study were obtained from BrainWeb at http://brainweb.bic.mni.mcgill.ca/brainweb/. Also, MR image dataset was obtained from the Open Access Series of Imaging Studies (OASIS) project at https://www.oasis-brains.org/.

## Appendix

### ADMM Optimization for Solving

In this appendix, we explain the procedure to derive the update rules (14)–(17) for (12). Each update rule can be obtained by minimizing (12) with respect to each variable one by one.

First, (14) can be obtained by the following partial derivative:

(A.1) ∂L∂f=RΩTRΩf−RΩf0′−GTα+ρGTGf−ρGTx=RΩf−f0′−GTα+ρIf−ρGTx→0.

Thus, by solving (A.1) with respect to **f**, we obtain

(A.2) RΩ+ρIf=f0′−GTα+ρGTx,f=RΩ+ρI−1RΩf0′+GTα+ρx.

Similar to the case with **f**, the update rule (15) for **x** can be obtained from

(A.3) ∂L∂x=ϵ∑i=1Nx−mi+vi−LTz+α+RΓ¯Tγ+ρLTLx−LTy+ρx−Gf+ρRΓ¯x→0.

For (16), the following problem with respect to **Y** = mat(**y**) is solved:

(A.4) minimizeY λTVY1,2+ρ2Y−Lx+1ρmatzF2.

The update rules of **y** and **z** for solving (A.4) are given by ADMM as

(A.5) yk+1=proxρ−1λTV·1,2Lxk+1−ρ−1zkzk+1=zk+ρyk+1−Lxk+1.

The proximal function for the *l*_1,2_-norm is defined as

(A.6) $$\begin{array}{c} {\left[\;prox_{\nu {\left\|\cdot \right\|}_{1,2}}\left(\;a\;\right)\;\right]}_{\iota }=a_{\iota }\circ \max\left(\;1-\frac{\nu }{{\left\|a_{\iota }\right\|}_{2}},0\;\right), \end{array}$$

where **a** = [**a**_1_, **a**_2_, ⋯, **a**_*I*_]^T^. By applying **w**^*k*^ = **L****x**^*k*+1^ − *ρ*^−1^**z**^*k*^ to the notations above, the update rule(16) can be obtained by

(A.7) yk+1s=proxρ−1λTV·1,2wsk=max⁡1−λTVρwsk2−1,0wsk,

where **w**_*s*_^*k*^ = [*w*_*s*1_^*k*^, *w*_*s*2_^*k*^, *w*_*s*3_^*k*^]^T^.

Finally, the subproblem with respect to **m**_*i*_ = vec(*M*_*i*_), denoted by

(A.8) minimizemi λLRNMii∗+ϵ2x−mi+vi22,

is solved to obtain (17). The optimal solution is given by singular value thresholding [25, 35]:

(A.9) $$\begin{array}{c} M_{i}^{k+1}={\text{fold}}_{i}\left[\;{SVT}_{\lambda_{LR}/N\epsilon }\left(\;{\text{unfold}}_{j}\left(\;X^{k+1}+V_{i}^{k}\;\right)\;\right)\;\right]. \end{array}$$

## Supplementary Materials

Supplementary Materials There are results of the preliminary experiments using 2D simple synthetic images. We simulated 16 images of variational patterns to be restored.* Experimental settings:* We compared the performance of the proposed method (LRTVG) with the Gerchberg algorithm [7], TV regularized super-resolution [22], and LRTV [23]. The ground truth synthetic image is first blurred toward the row-direction with a rectangular profile spectrum. Two blurred images were obtained for each ground truth by cutting off 65% and 85% of the spectrum toward row-direction. Each blurred image was also contaminated with Gaussian noise (3%-noise level) or free of noise (0%-noise level). Accordingly, four patterns of the observations are obtained from two blur kernels and two noise levels. The images are then reconstructed from four patterns of the observations using each method, and the reconstructed images are evaluated with both PSNR and SSIM [60]. We also evaluated the performances of Gerchberg method and the proposed method with respect to the accuracy of the region Γ. The experiment was conducted by making Γ redundant from the true boundary. The distance from the true boundary is changed from 0 to 10.* About files:* there are six PDF files in the Supplementary Materials S1–S6. The four files named S1-S4 include the illustrations of results of 16 synthetic images ((A)-(P)). Each of the four files includes results of four of the 16 images. The file named S5 includes the PSNR and SSIM results of the respective 16 images, (A)-(P). The file named S6 includes the PSNR and SSIM results of each image when the contour of Γ is redundant from the true boundary. The results of the cases when distances from the true boundary, dist., equal 0, 4, and 8 are additionally plotted on the figures. Also, the folder named S7 includes the PSNR results with respect to *λ*_TV_ and *λ*_LR_ for variational images in Table 1. Yellow/blue colors mean high/low PSNR values.
