# Supplementary figures and images for: Super-Resolution of Magnetic Resonance Images via Convex Optimization with Local and Global Prior Regularization and Spectrum Fitting
Source: Int J Biomed Imaging. 2018 Sep 2;2018:9262847. doi: 10.1155/2018/9262847 (PMC6139240; doi:10.1155/2018/9262847)

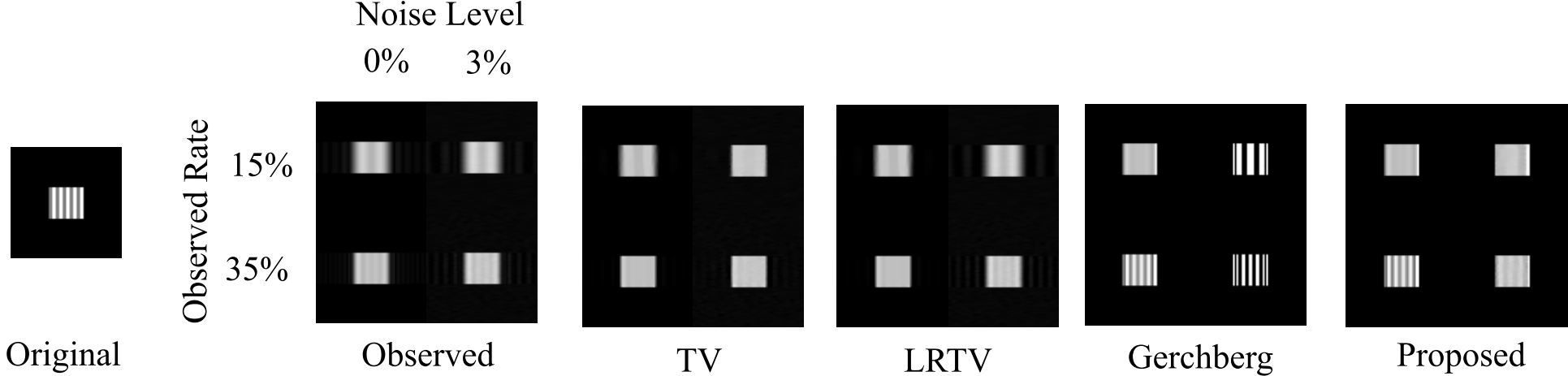

(A)

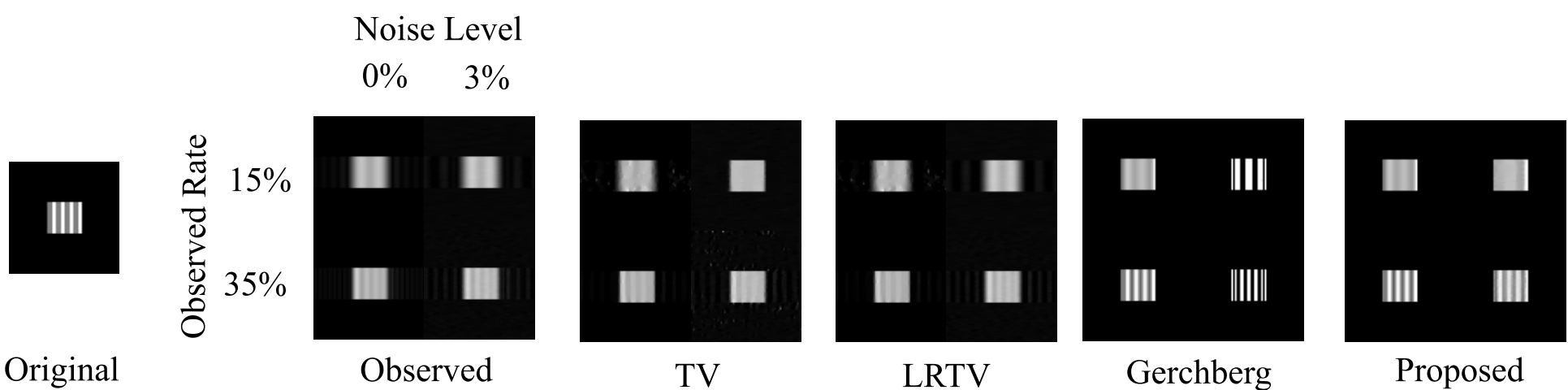

(B)

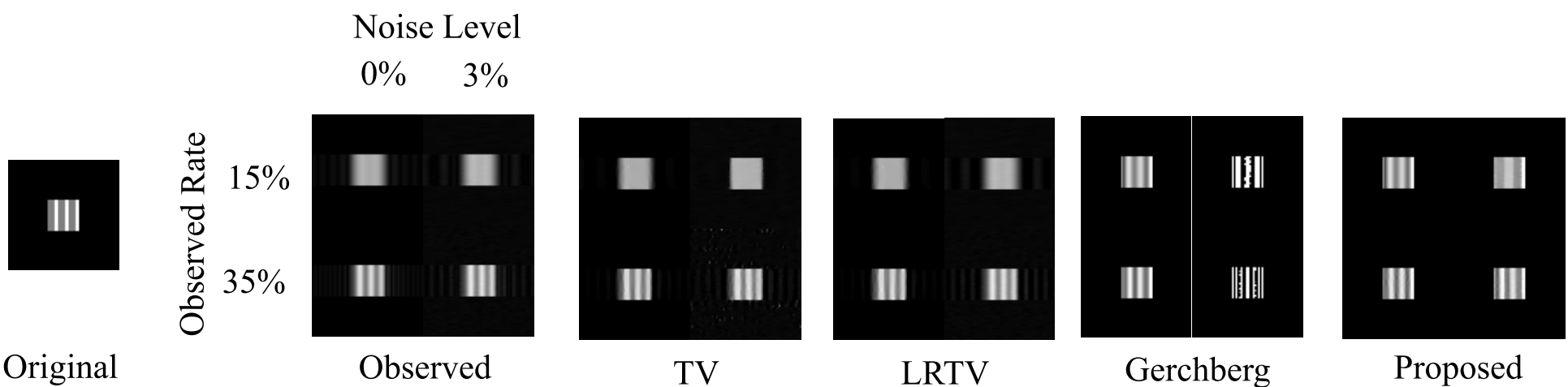

(C)

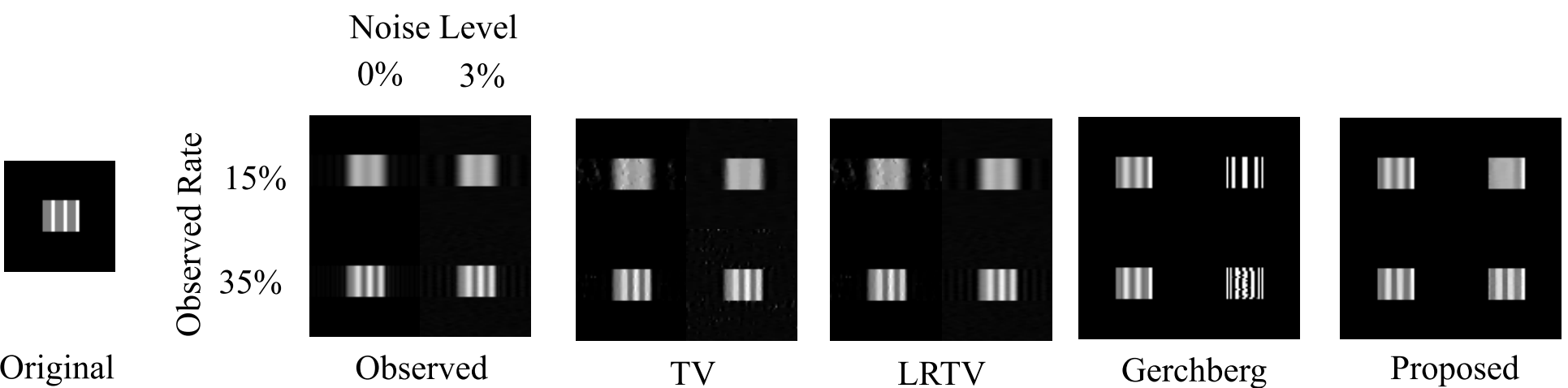

(D)

Supplement: Supplementary Materials — There are results of the preliminary experiments using 2D simple synthetic images. We simulated 16 images of variational patterns to be restored. Experimental settings: We compared the performance of the proposed method (LRTVG) with the Gerchberg algorithm [7], TV regularized super-resolution [22], and LRTV [23]. The ground truth synthetic image is first blurred toward the row-direction with a rectangular profile spectrum. Two blurred images were obtained for each ground truth by cutting off 65% and 85% of the spectrum toward row-direction. Each blurred image was also contaminated with Gaussian noise (3%-noise level) or free of noise (0%-noise level). Accordingly, four patterns of the observations are obtained from two blur kernels and two noise levels. The images are then reconstructed from four patterns of the observations using each method, and the reconstructed images are evaluated with both PSNR and SSIM [60]. We also evaluated the performances of Gerchberg method and the proposed method with respect to the accuracy of the region Γ. The experiment was conducted by making Γ redundant from the true boundary. The distance from the true boundary is changed from 0 to 10. About files: there are six PDF files in the Supplementary Materials S1–S6. The four files named S1-S4 include the illustrations of results of 16 synthetic images ((A)-(P)). Each of the four files includes results of four of the 16 images. The file named S5 includes the PSNR and SSIM results of the respective 16 images, (A)-(P). The file named S6 includes the PSNR and SSIM results of each image when the contour of Γ is redundant from the true boundary. The results of the cases when distances from the true boundary, dist., equal 0, 4, and 8 are additionally plotted on the figures. Also, the folder named S7 includes the PSNR results with respect to λTV and λLR for variational images in Table 1. Yellow/blue colors mean high/low PSNR values. [file 9262847.f1.zip › 9262847.f1.pdf]

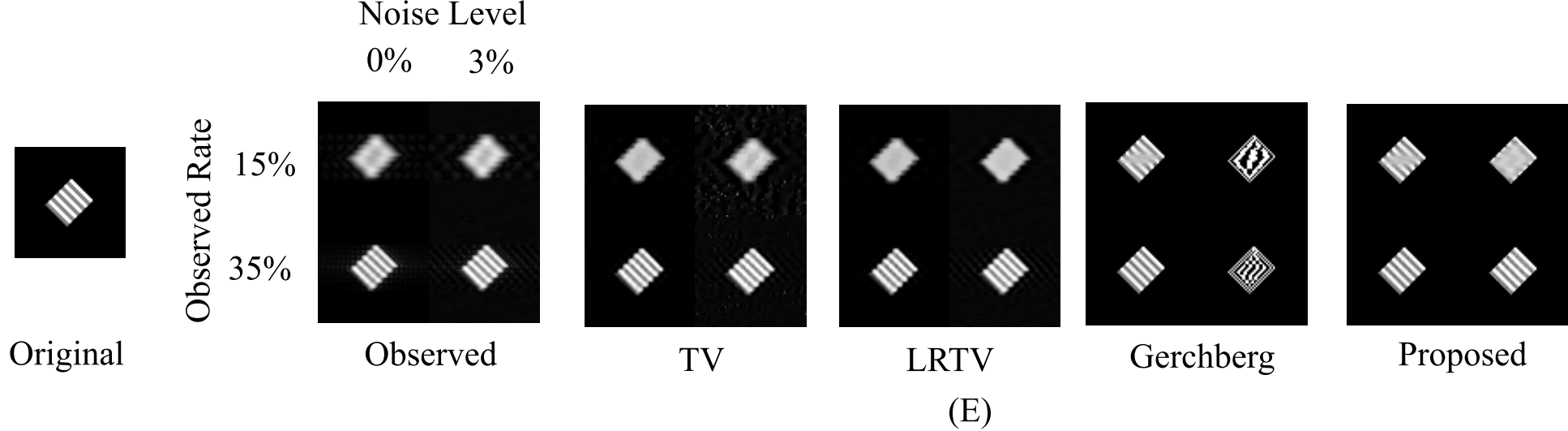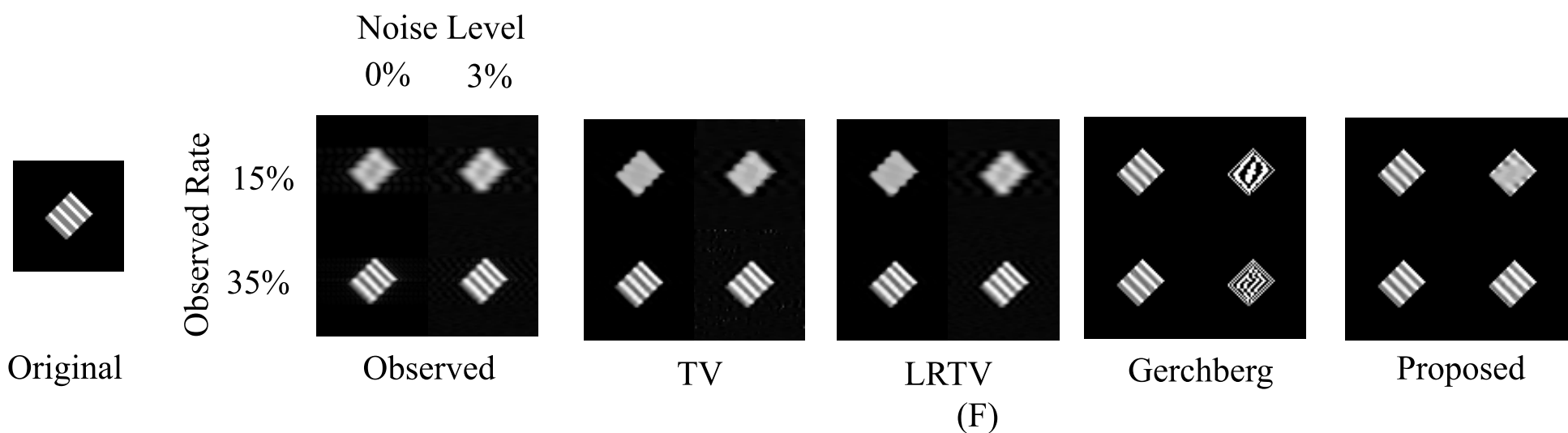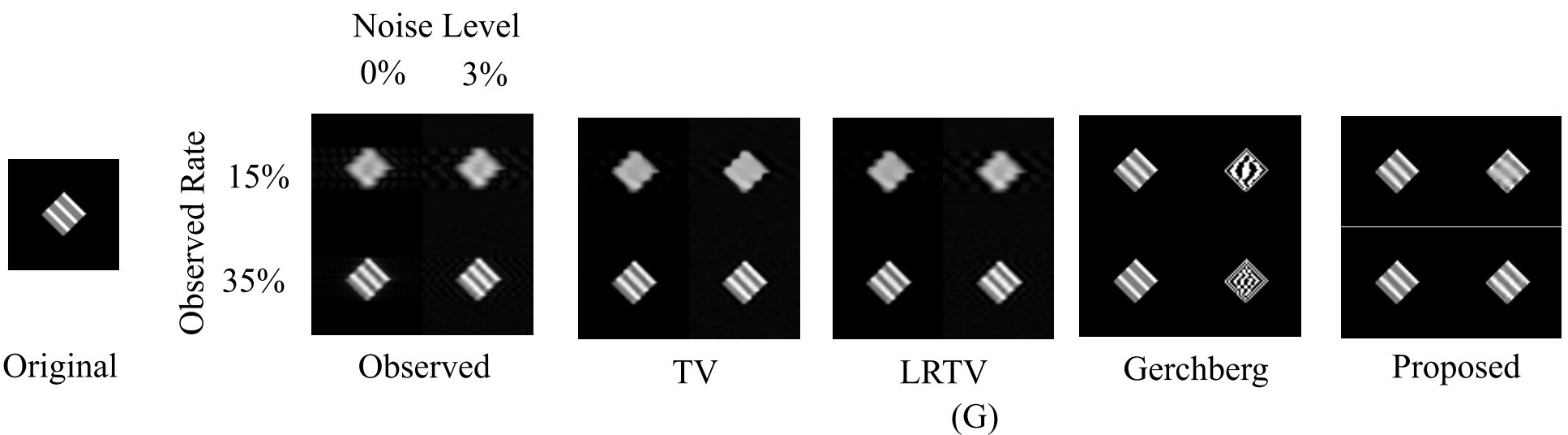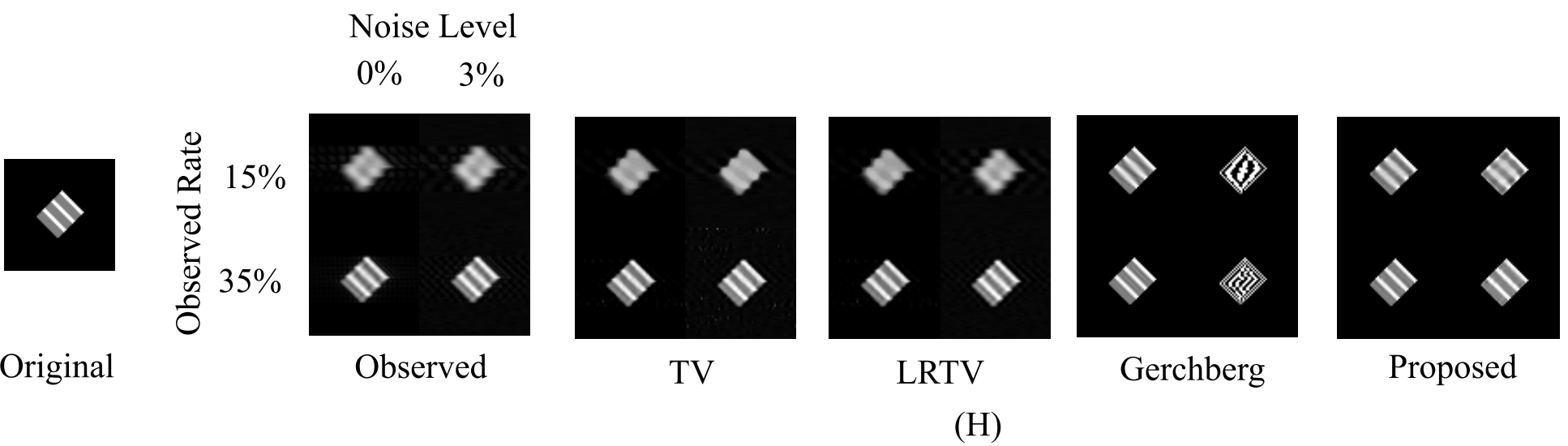

Supplement: Supplementary Materials — There are results of the preliminary experiments using 2D simple synthetic images. We simulated 16 images of variational patterns to be restored. Experimental settings: We compared the performance of the proposed method (LRTVG) with the Gerchberg algorithm [7], TV regularized super-resolution [22], and LRTV [23]. The ground truth synthetic image is first blurred toward the row-direction with a rectangular profile spectrum. Two blurred images were obtained for each ground truth by cutting off 65% and 85% of the spectrum toward row-direction. Each blurred image was also contaminated with Gaussian noise (3%-noise level) or free of noise (0%-noise level). Accordingly, four patterns of the observations are obtained from two blur kernels and two noise levels. The images are then reconstructed from four patterns of the observations using each method, and the reconstructed images are evaluated with both PSNR and SSIM [60]. We also evaluated the performances of Gerchberg method and the proposed method with respect to the accuracy of the region Γ. The experiment was conducted by making Γ redundant from the true boundary. The distance from the true boundary is changed from 0 to 10. About files: there are six PDF files in the Supplementary Materials S1–S6. The four files named S1-S4 include the illustrations of results of 16 synthetic images ((A)-(P)). Each of the four files includes results of four of the 16 images. The file named S5 includes the PSNR and SSIM results of the respective 16 images, (A)-(P). The file named S6 includes the PSNR and SSIM results of each image when the contour of Γ is redundant from the true boundary. The results of the cases when distances from the true boundary, dist., equal 0, 4, and 8 are additionally plotted on the figures. Also, the folder named S7 includes the PSNR results with respect to λTV and λLR for variational images in Table 1. Yellow/blue colors mean high/low PSNR values. [file 9262847.f1.zip › 9262847.f2.pdf]

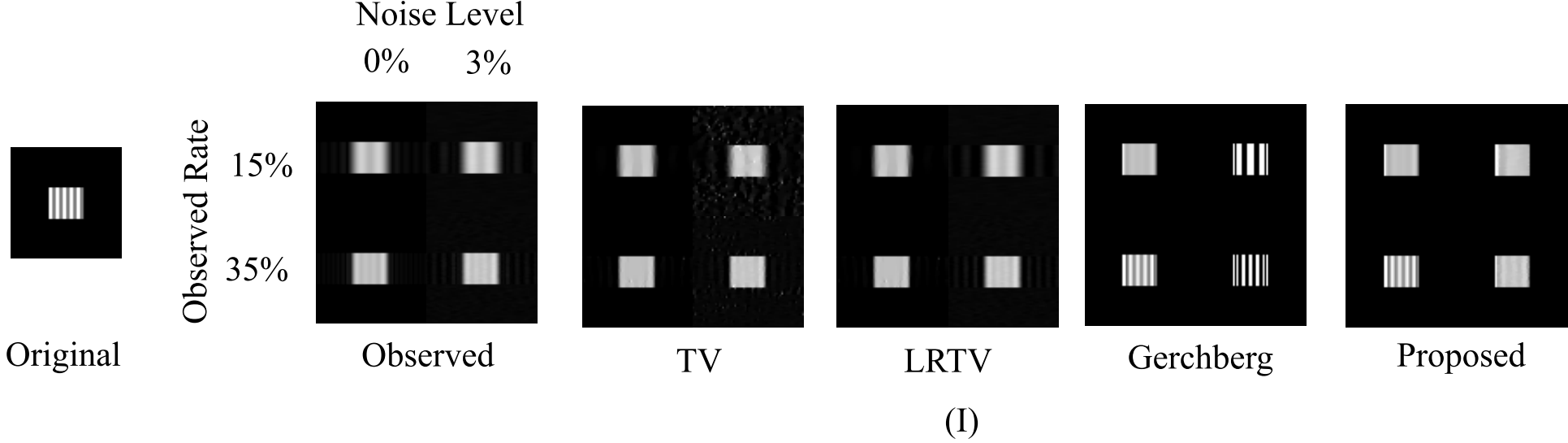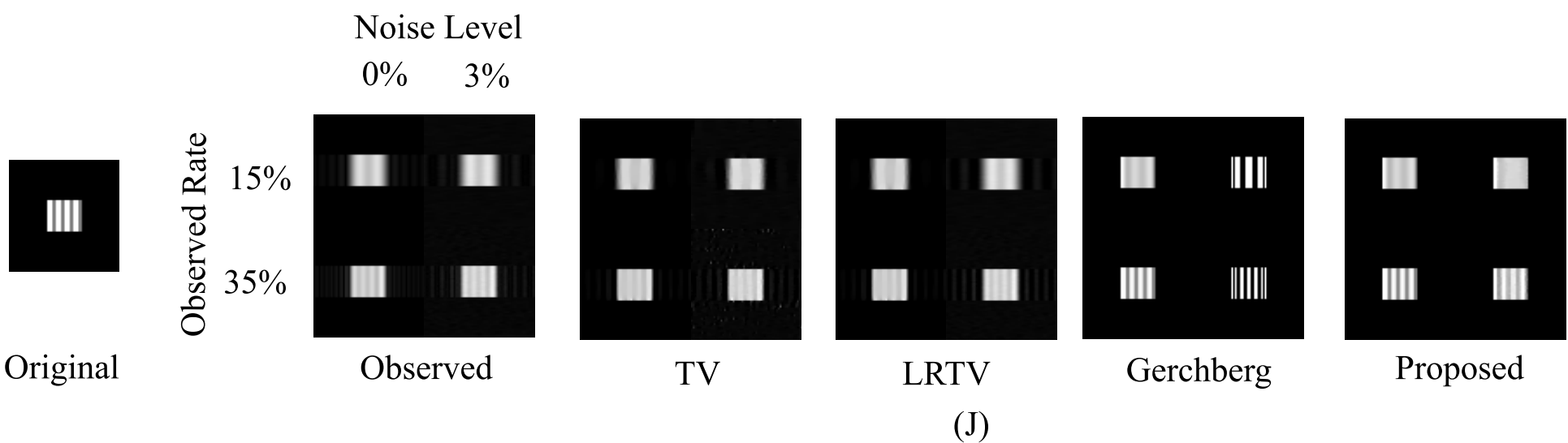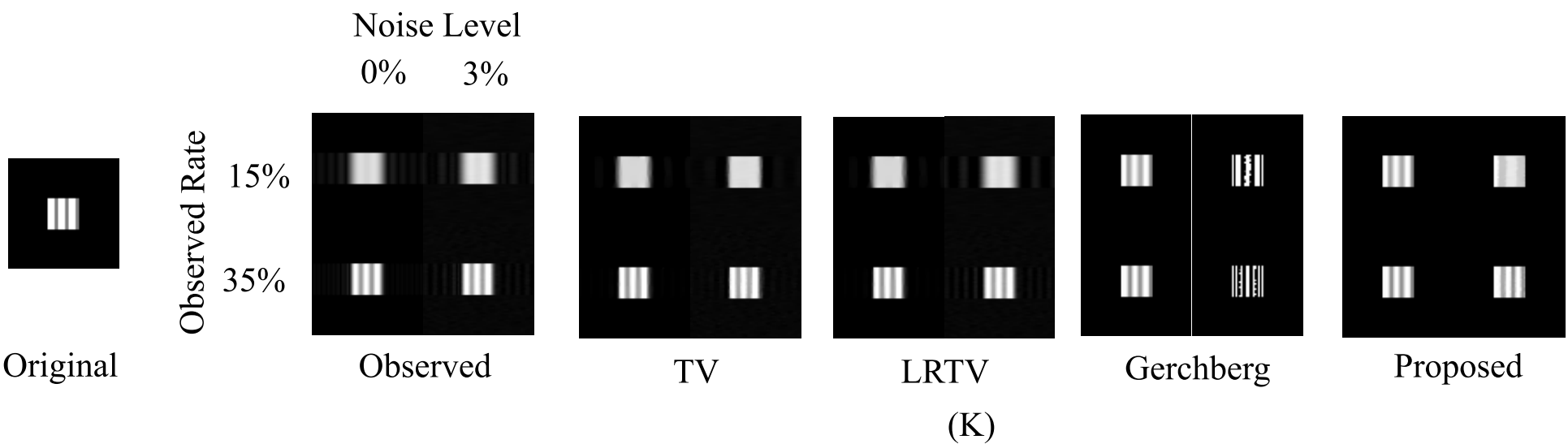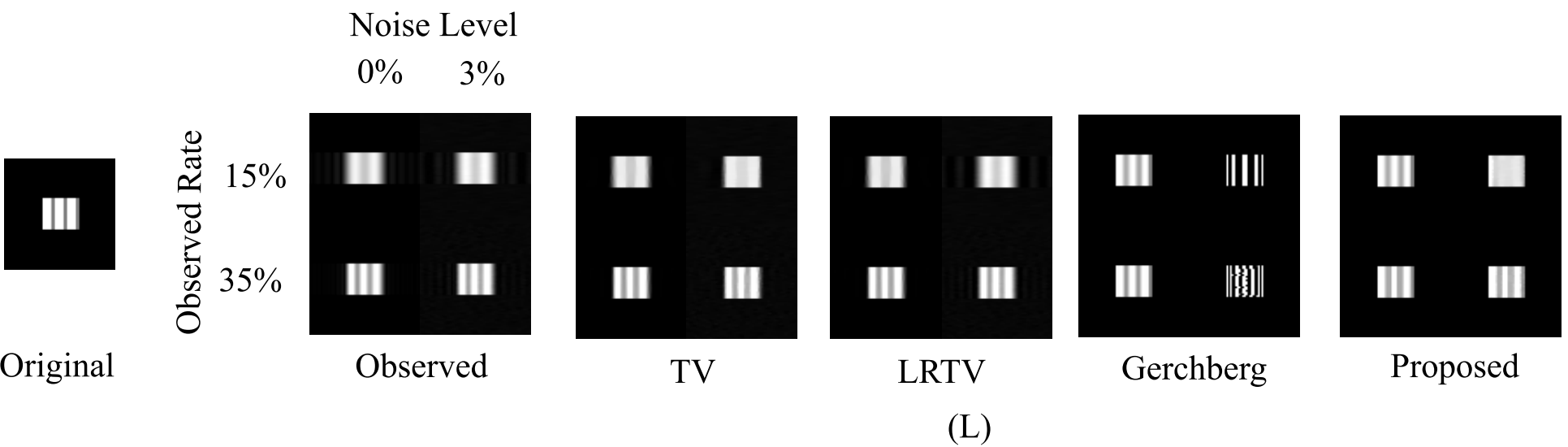

Supplement: Supplementary Materials — There are results of the preliminary experiments using 2D simple synthetic images. We simulated 16 images of variational patterns to be restored. Experimental settings: We compared the performance of the proposed method (LRTVG) with the Gerchberg algorithm [7], TV regularized super-resolution [22], and LRTV [23]. The ground truth synthetic image is first blurred toward the row-direction with a rectangular profile spectrum. Two blurred images were obtained for each ground truth by cutting off 65% and 85% of the spectrum toward row-direction. Each blurred image was also contaminated with Gaussian noise (3%-noise level) or free of noise (0%-noise level). Accordingly, four patterns of the observations are obtained from two blur kernels and two noise levels. The images are then reconstructed from four patterns of the observations using each method, and the reconstructed images are evaluated with both PSNR and SSIM [60]. We also evaluated the performances of Gerchberg method and the proposed method with respect to the accuracy of the region Γ. The experiment was conducted by making Γ redundant from the true boundary. The distance from the true boundary is changed from 0 to 10. About files: there are six PDF files in the Supplementary Materials S1–S6. The four files named S1-S4 include the illustrations of results of 16 synthetic images ((A)-(P)). Each of the four files includes results of four of the 16 images. The file named S5 includes the PSNR and SSIM results of the respective 16 images, (A)-(P). The file named S6 includes the PSNR and SSIM results of each image when the contour of Γ is redundant from the true boundary. The results of the cases when distances from the true boundary, dist., equal 0, 4, and 8 are additionally plotted on the figures. Also, the folder named S7 includes the PSNR results with respect to λTV and λLR for variational images in Table 1. Yellow/blue colors mean high/low PSNR values. [file 9262847.f1.zip › 9262847.f3.pdf]

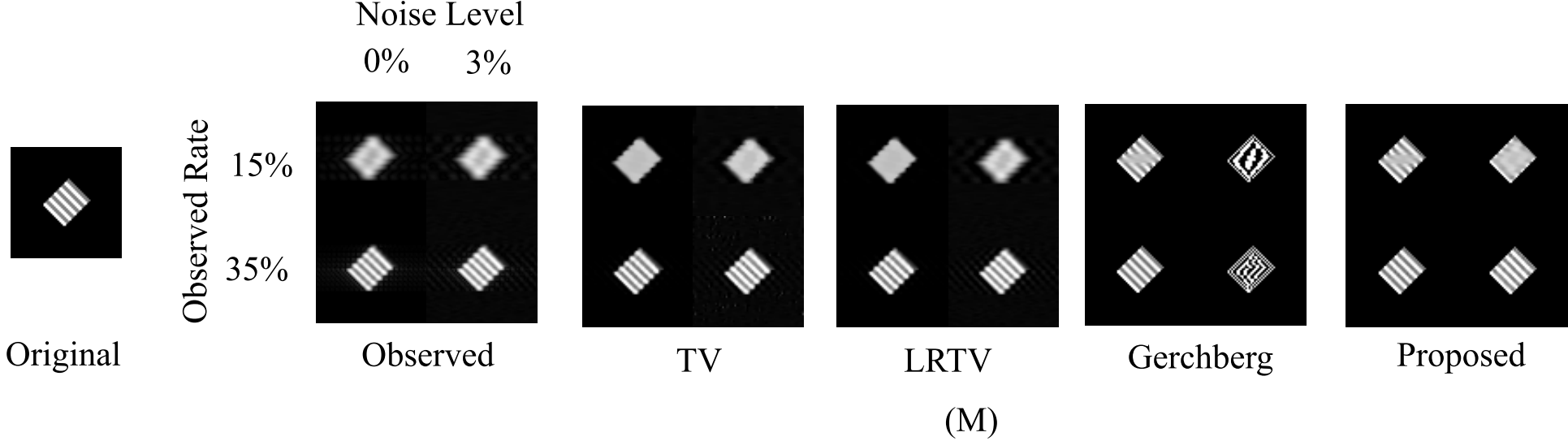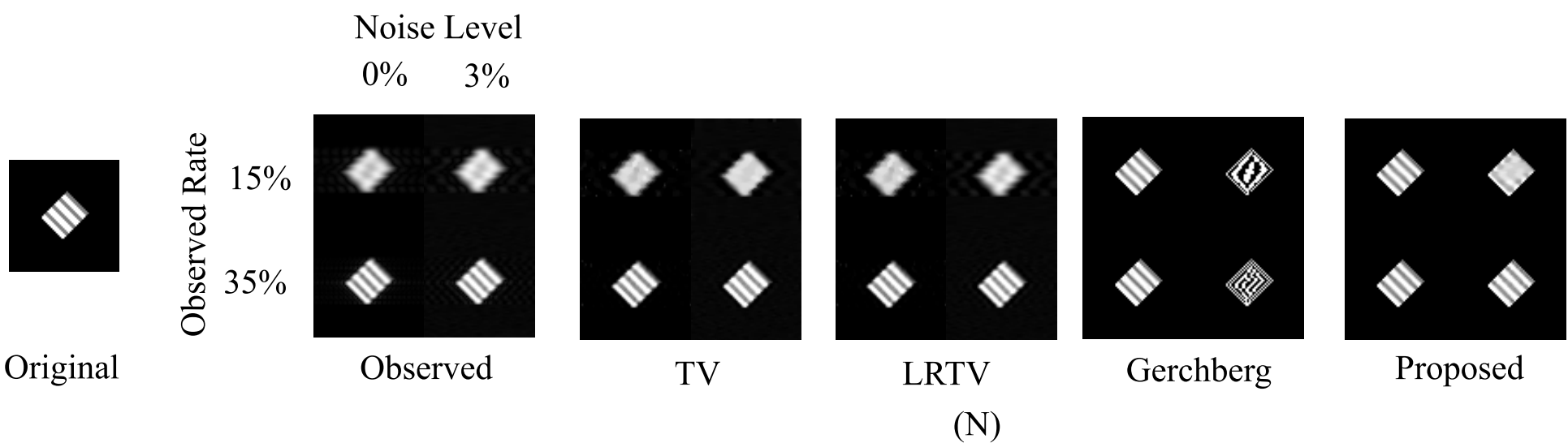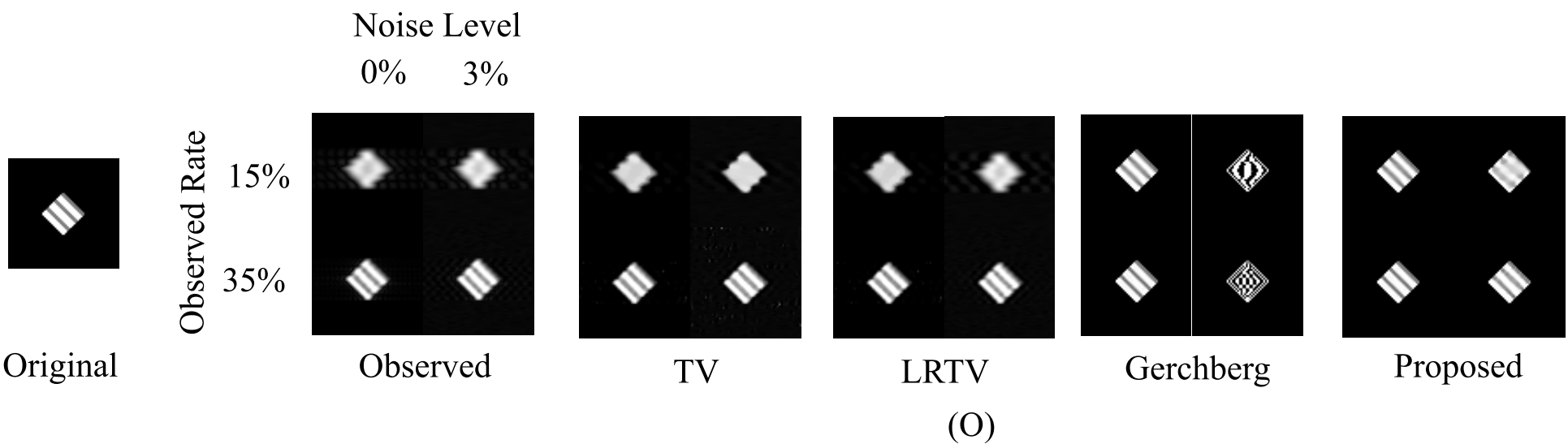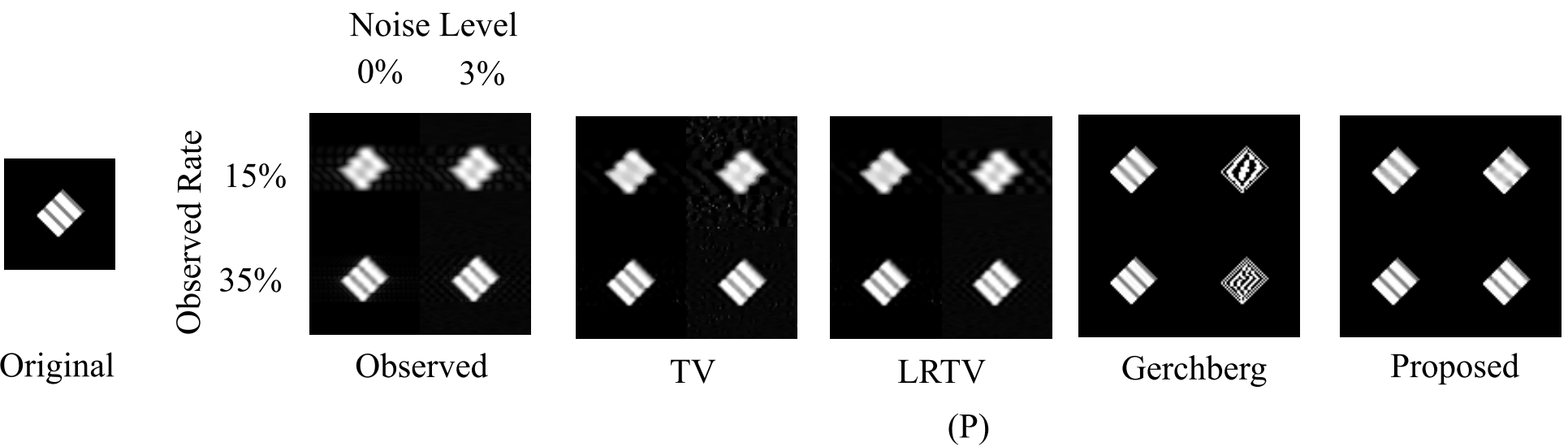

Supplement: Supplementary Materials — There are results of the preliminary experiments using 2D simple synthetic images. We simulated 16 images of variational patterns to be restored. Experimental settings: We compared the performance of the proposed method (LRTVG) with the Gerchberg algorithm [7], TV regularized super-resolution [22], and LRTV [23]. The ground truth synthetic image is first blurred toward the row-direction with a rectangular profile spectrum. Two blurred images were obtained for each ground truth by cutting off 65% and 85% of the spectrum toward row-direction. Each blurred image was also contaminated with Gaussian noise (3%-noise level) or free of noise (0%-noise level). Accordingly, four patterns of the observations are obtained from two blur kernels and two noise levels. The images are then reconstructed from four patterns of the observations using each method, and the reconstructed images are evaluated with both PSNR and SSIM [60]. We also evaluated the performances of Gerchberg method and the proposed method with respect to the accuracy of the region Γ. The experiment was conducted by making Γ redundant from the true boundary. The distance from the true boundary is changed from 0 to 10. About files: there are six PDF files in the Supplementary Materials S1–S6. The four files named S1-S4 include the illustrations of results of 16 synthetic images ((A)-(P)). Each of the four files includes results of four of the 16 images. The file named S5 includes the PSNR and SSIM results of the respective 16 images, (A)-(P). The file named S6 includes the PSNR and SSIM results of each image when the contour of Γ is redundant from the true boundary. The results of the cases when distances from the true boundary, dist., equal 0, 4, and 8 are additionally plotted on the figures. Also, the folder named S7 includes the PSNR results with respect to λTV and λLR for variational images in Table 1. Yellow/blue colors mean high/low PSNR values. [file 9262847.f1.zip › 9262847.f4.pdf]

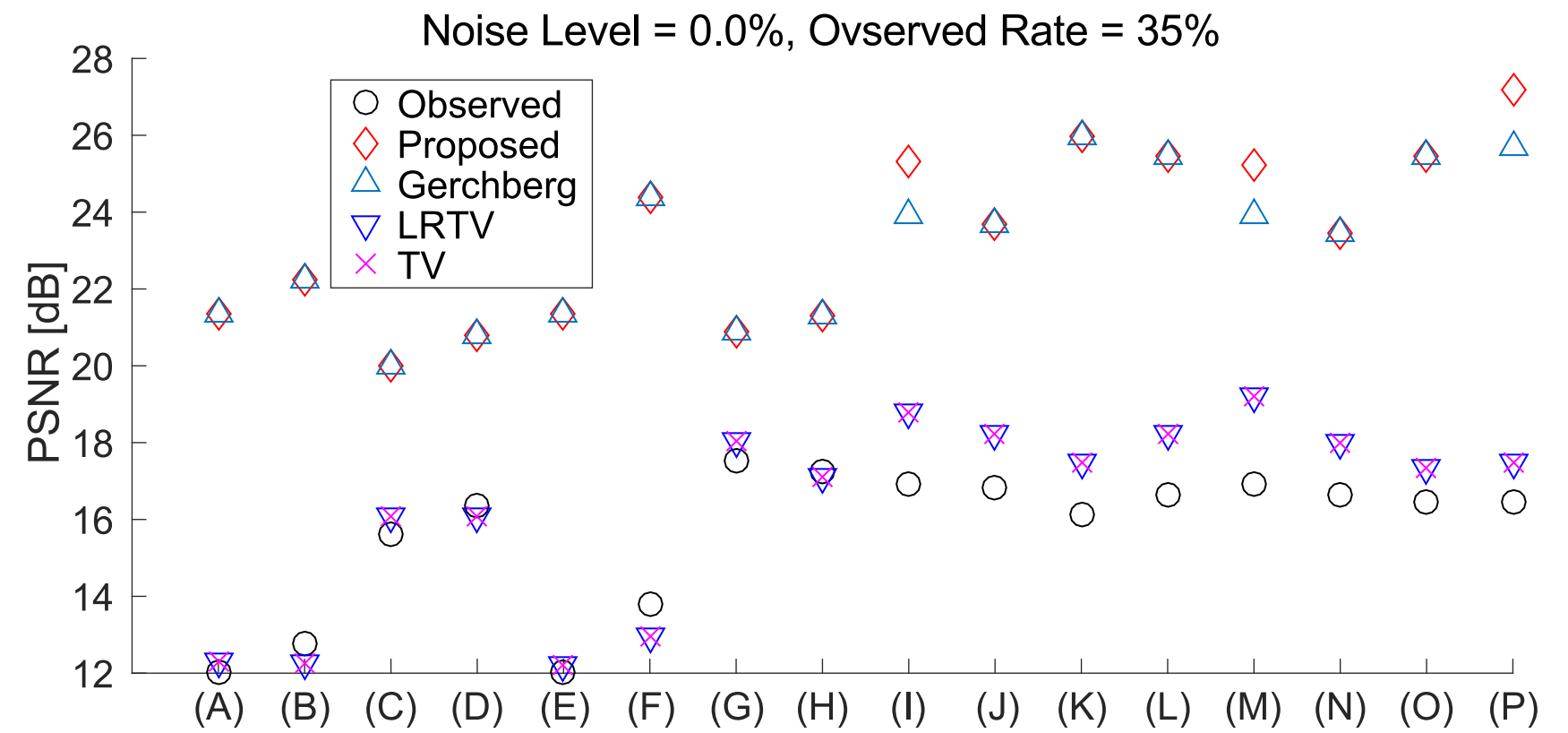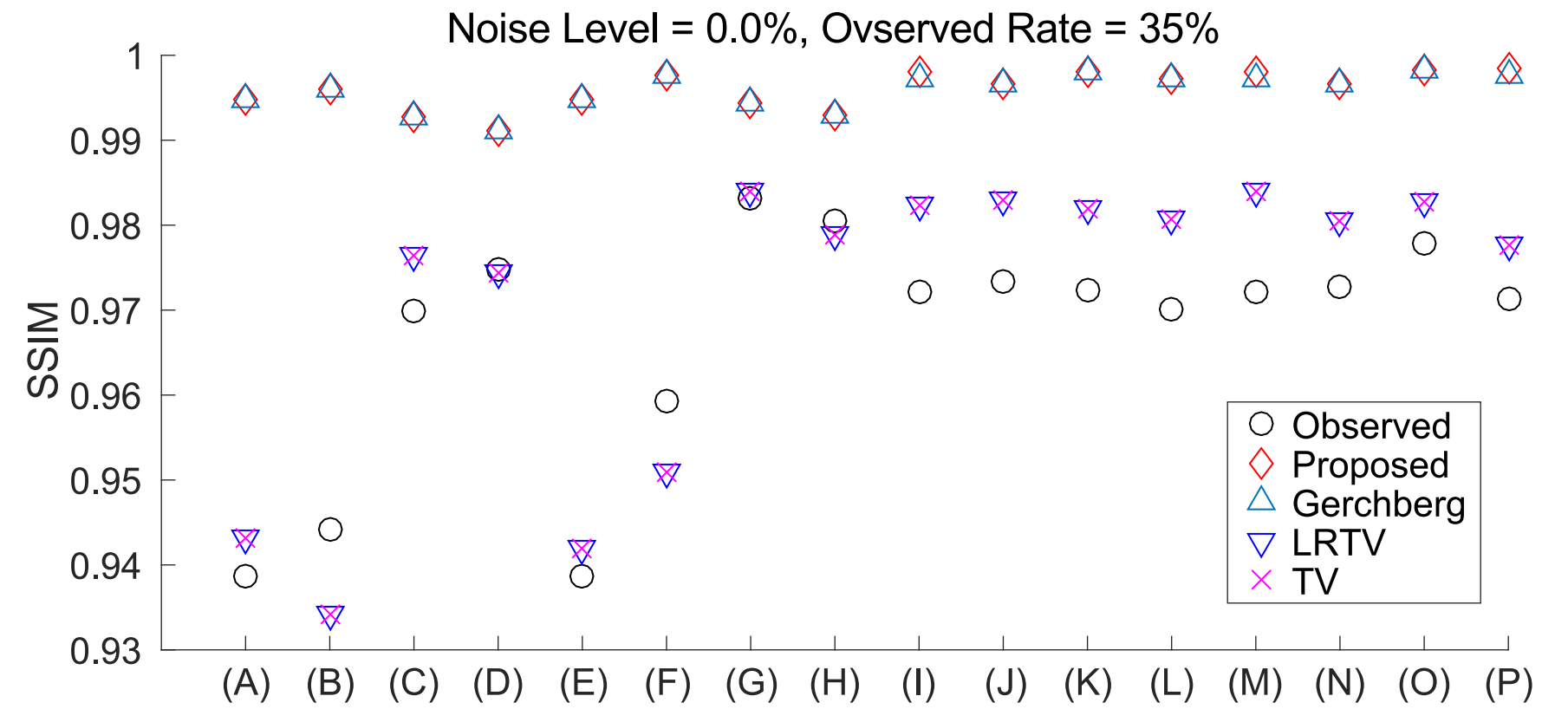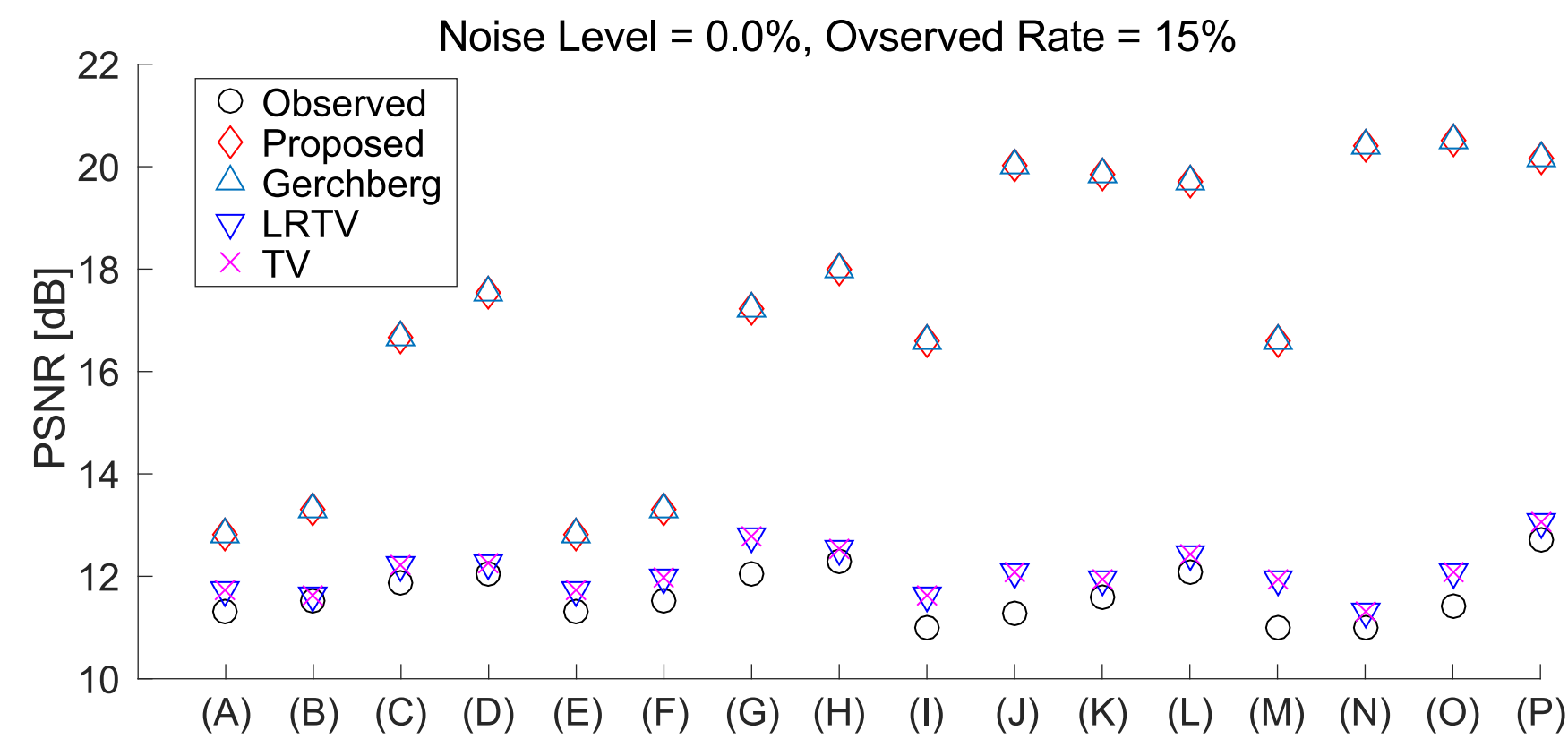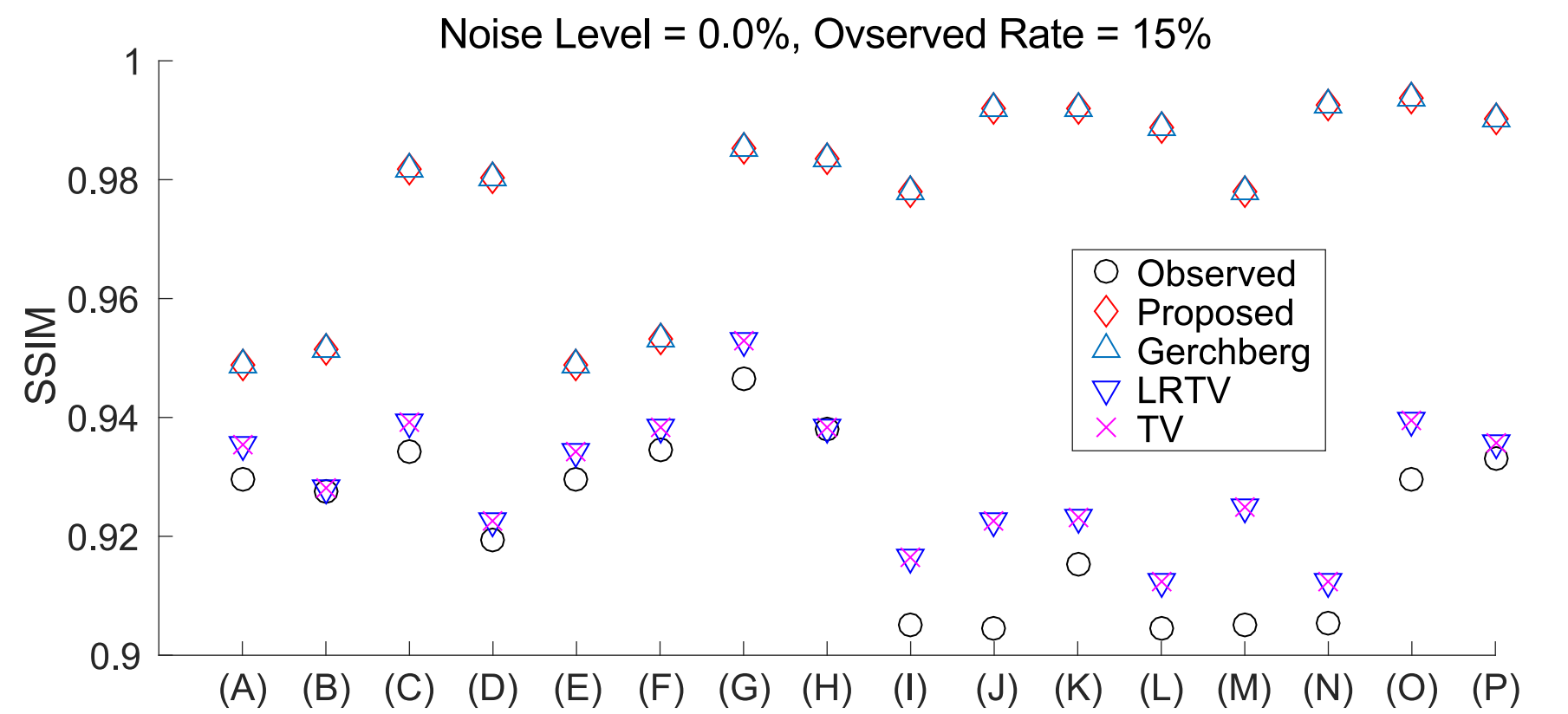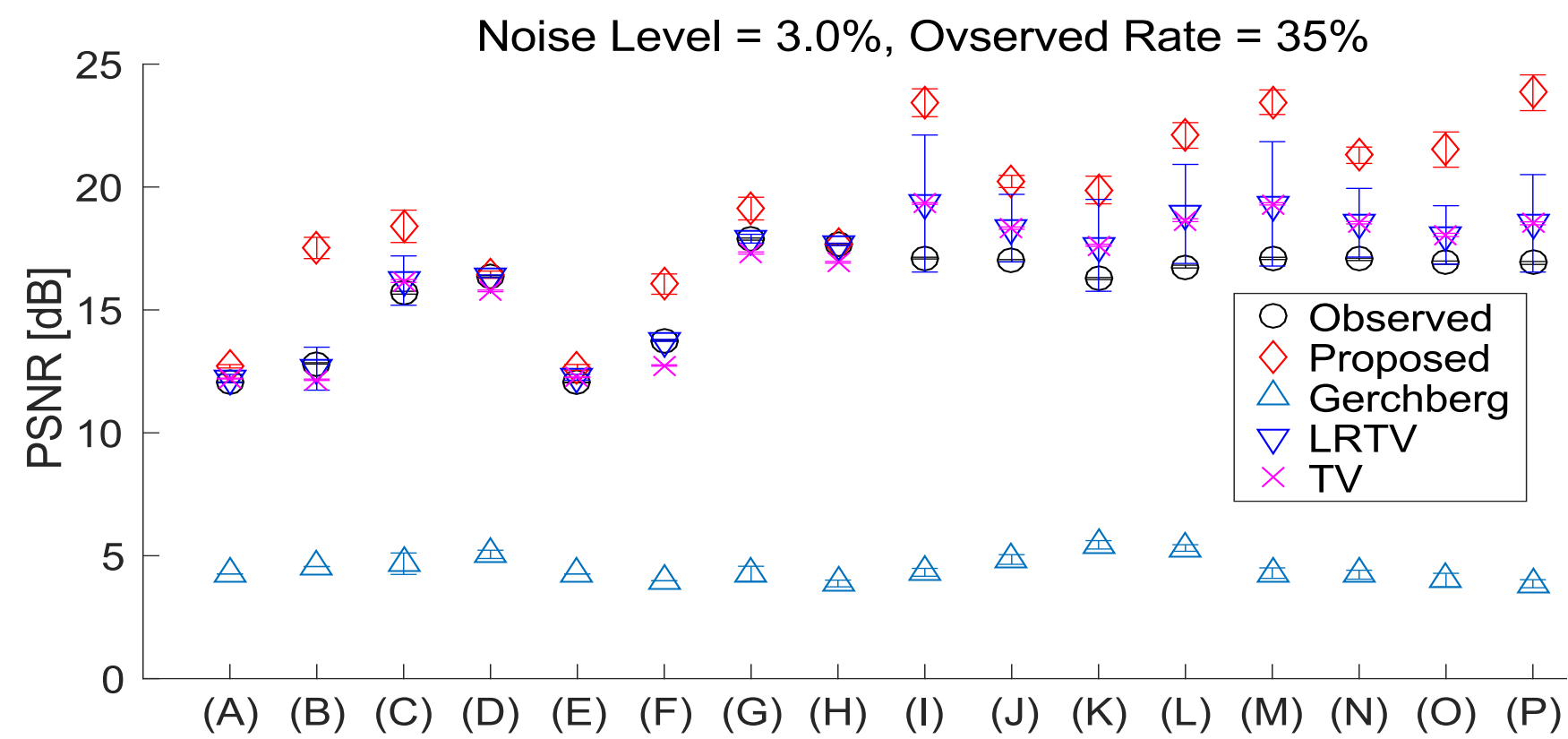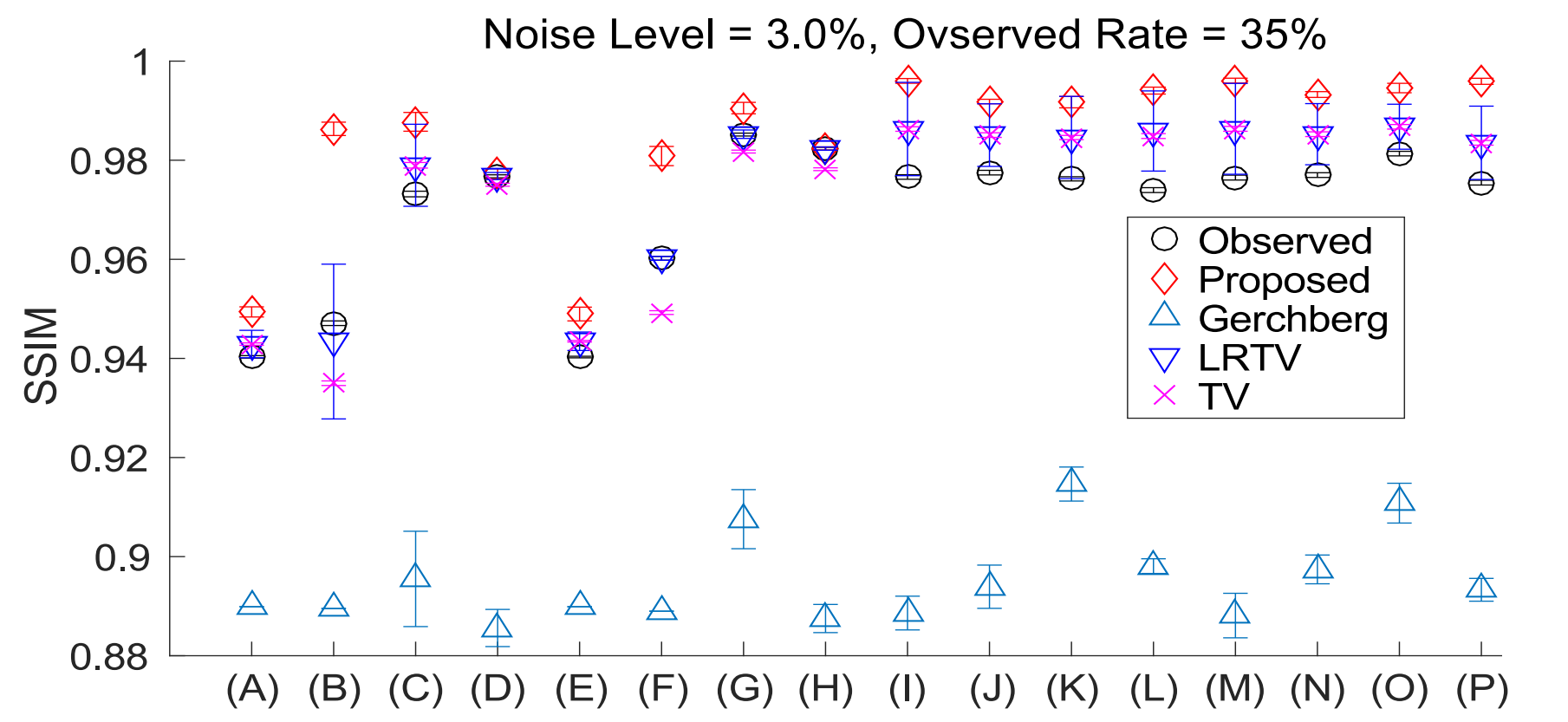

Supplement: Supplementary Materials — There are results of the preliminary experiments using 2D simple synthetic images. We simulated 16 images of variational patterns to be restored. Experimental settings: We compared the performance of the proposed method (LRTVG) with the Gerchberg algorithm [7], TV regularized super-resolution [22], and LRTV [23]. The ground truth synthetic image is first blurred toward the row-direction with a rectangular profile spectrum. Two blurred images were obtained for each ground truth by cutting off 65% and 85% of the spectrum toward row-direction. Each blurred image was also contaminated with Gaussian noise (3%-noise level) or free of noise (0%-noise level). Accordingly, four patterns of the observations are obtained from two blur kernels and two noise levels. The images are then reconstructed from four patterns of the observations using each method, and the reconstructed images are evaluated with both PSNR and SSIM [60]. We also evaluated the performances of Gerchberg method and the proposed method with respect to the accuracy of the region Γ. The experiment was conducted by making Γ redundant from the true boundary. The distance from the true boundary is changed from 0 to 10. About files: there are six PDF files in the Supplementary Materials S1–S6. The four files named S1-S4 include the illustrations of results of 16 synthetic images ((A)-(P)). Each of the four files includes results of four of the 16 images. The file named S5 includes the PSNR and SSIM results of the respective 16 images, (A)-(P). The file named S6 includes the PSNR and SSIM results of each image when the contour of Γ is redundant from the true boundary. The results of the cases when distances from the true boundary, dist., equal 0, 4, and 8 are additionally plotted on the figures. Also, the folder named S7 includes the PSNR results with respect to λTV and λLR for variational images in Table 1. Yellow/blue colors mean high/low PSNR values. [file 9262847.f1.zip › 9262847.f5.pdf]

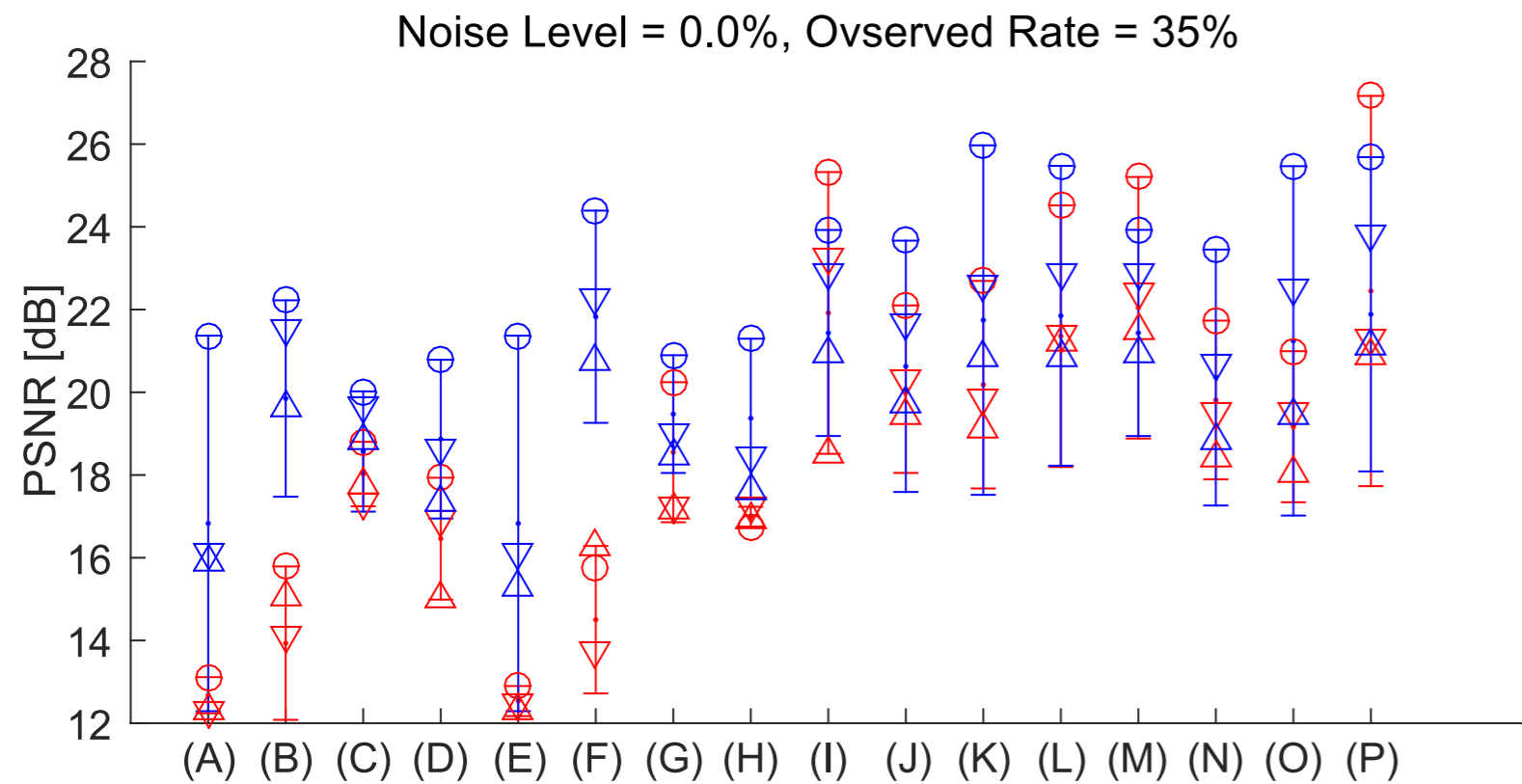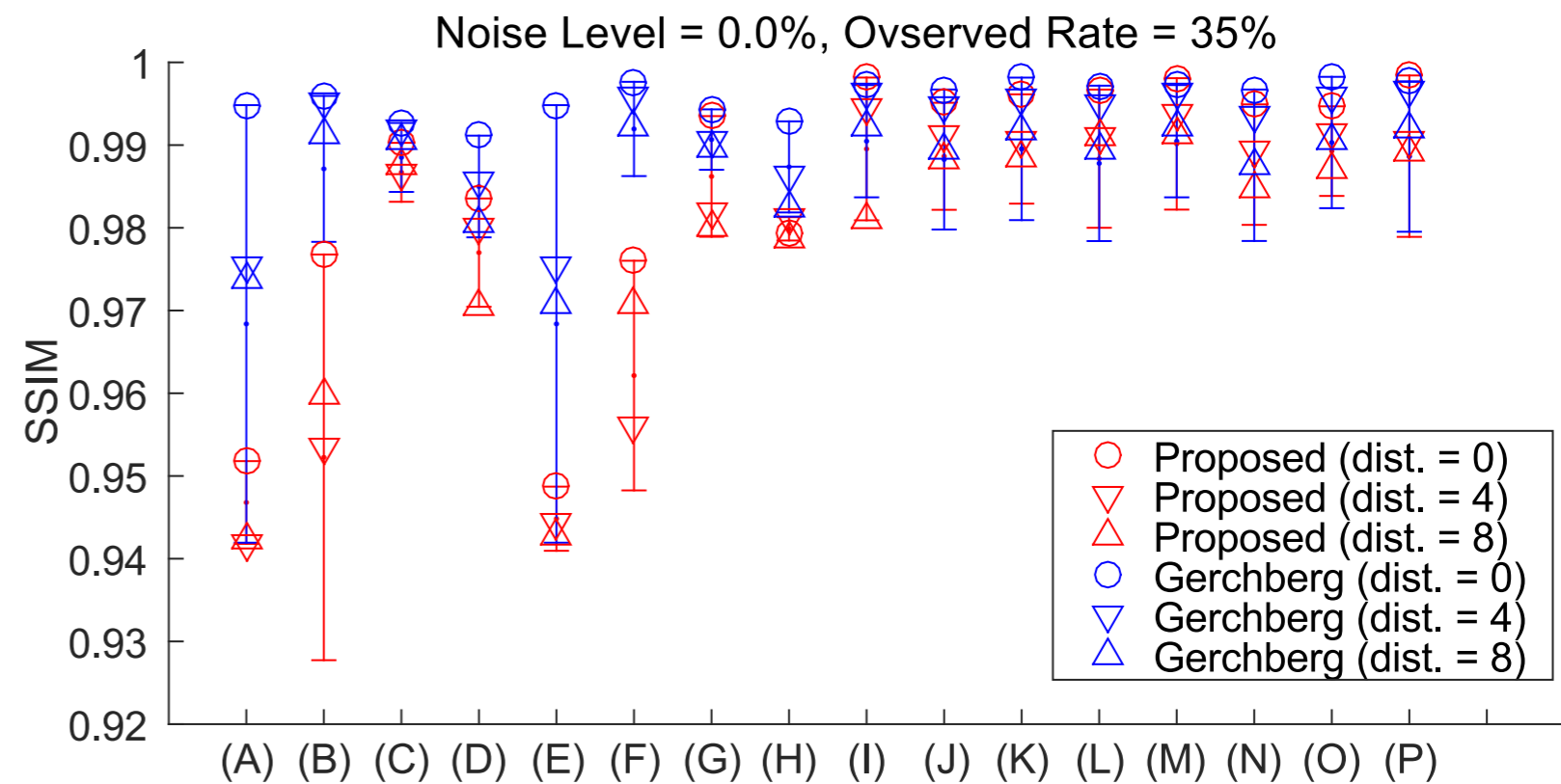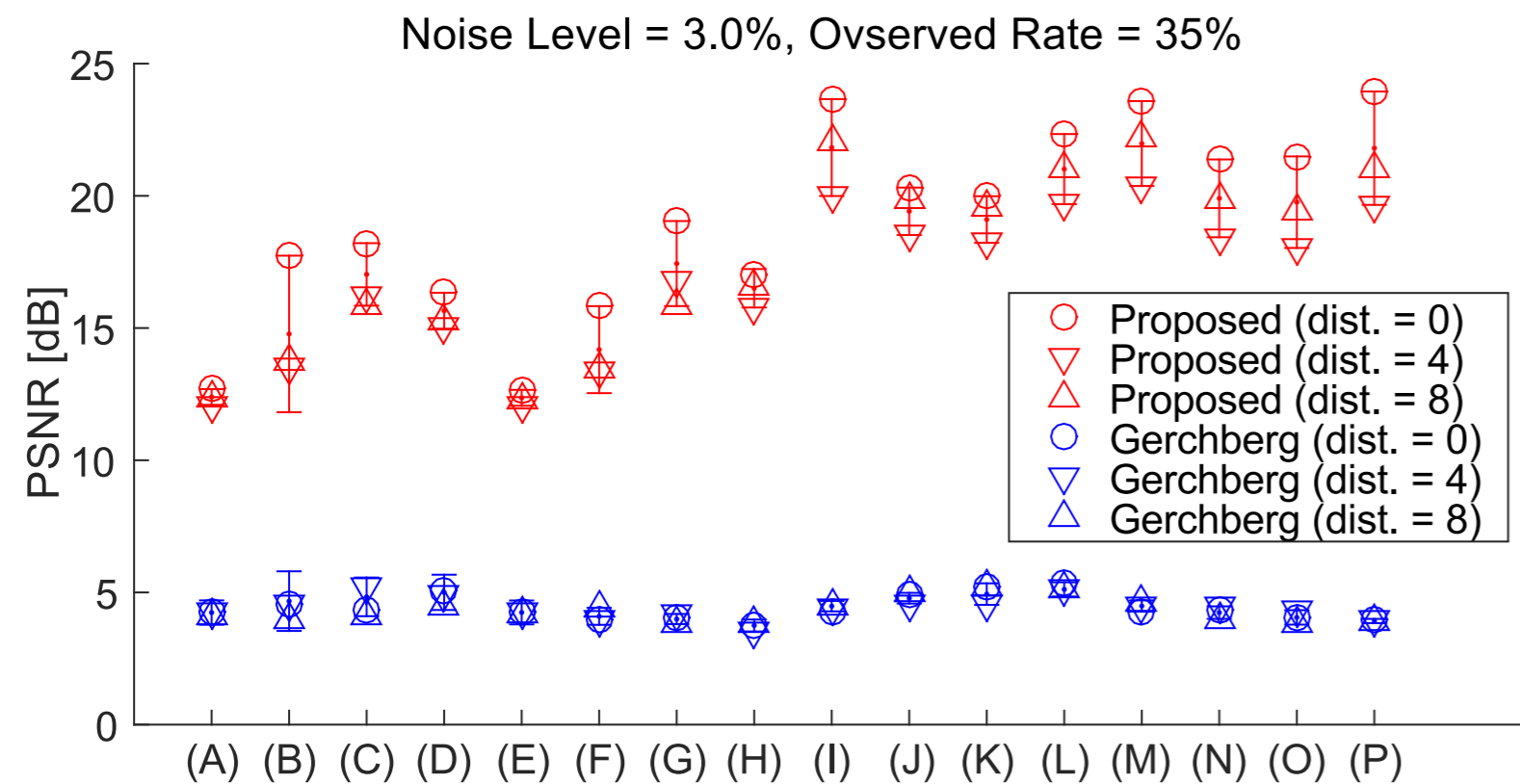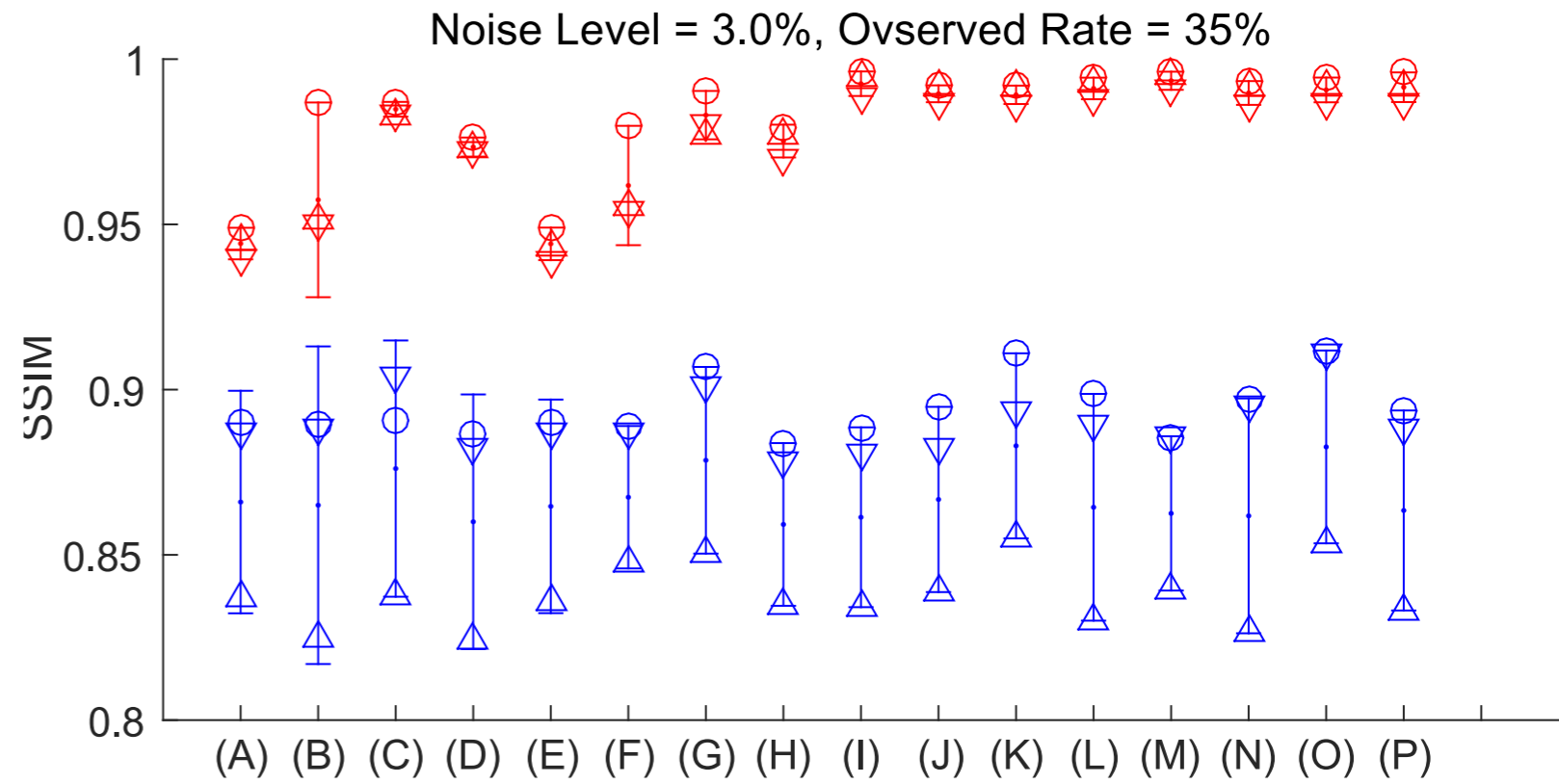

Supplement: Supplementary Materials — There are results of the preliminary experiments using 2D simple synthetic images. We simulated 16 images of variational patterns to be restored. Experimental settings: We compared the performance of the proposed method (LRTVG) with the Gerchberg algorithm [7], TV regularized super-resolution [22], and LRTV [23]. The ground truth synthetic image is first blurred toward the row-direction with a rectangular profile spectrum. Two blurred images were obtained for each ground truth by cutting off 65% and 85% of the spectrum toward row-direction. Each blurred image was also contaminated with Gaussian noise (3%-noise level) or free of noise (0%-noise level). Accordingly, four patterns of the observations are obtained from two blur kernels and two noise levels. The images are then reconstructed from four patterns of the observations using each method, and the reconstructed images are evaluated with both PSNR and SSIM [60]. We also evaluated the performances of Gerchberg method and the proposed method with respect to the accuracy of the region Γ. The experiment was conducted by making Γ redundant from the true boundary. The distance from the true boundary is changed from 0 to 10. About files: there are six PDF files in the Supplementary Materials S1–S6. The four files named S1-S4 include the illustrations of results of 16 synthetic images ((A)-(P)). Each of the four files includes results of four of the 16 images. The file named S5 includes the PSNR and SSIM results of the respective 16 images, (A)-(P). The file named S6 includes the PSNR and SSIM results of each image when the contour of Γ is redundant from the true boundary. The results of the cases when distances from the true boundary, dist., equal 0, 4, and 8 are additionally plotted on the figures. Also, the folder named S7 includes the PSNR results with respect to λTV and λLR for variational images in Table 1. Yellow/blue colors mean high/low PSNR values. [file 9262847.f1.zip › 9262847.f6.pdf]
